# Killer Cell Activating Receptor–Associated Protein Exacerbates Intestinal Inflammation Through Promoting Kynurenine–Aryl Hydrocarbon Receptor–Dependent Neutrophil Activation in Ulcerative Colitis

**DOI:** 10.1016/j.jcmgh.2026.101906

**Published:** 2026-09-24

**Authors:** Guiyuan Jin, Fengqin Zhu, Fengxian Dai, Yan Wang, Yun Chen, Yonghong Yang, Zhanju Liu, Guangxi Zhou

**Affiliations:** 1 Taishan Scholars Laboratory, Medical Research Center, Affiliated Hospital of Jining Medical University, Jining, Shandong Province, China; 2 Department of Gastroenterology, Affiliated Hospital of Jining Medical University, Jining, Shandong Province, China; 3 Center for Inflammatory Bowel Disease Research and Department of Gastroenterology, Affiliated Suzhou Hospital of Nanjing Medical University, Suzhou Municipal Hospital, Gusu School, Nanjing Medical University, Suzhou, China; 4 Department of Gastroenterology, Shanghai Tenth People’s Hospital of Tongji University, Shanghai, China

**Keywords:** Intestinal Epithelial Barrier, KARAP, Kynurenine, Neutrophils, Ulcerative Colitis

## Abstract

**Background & Aims:**

Killer cell activating receptor–associated protein, also known as TYRO protein tyrosine kinase binding protein, has been reported to participate in the regulation of autoimmunity and inflammation. However, the underlying mechanisms involved in intestinal mucosal inflammation in ulcerative colitis remain obscure.

**Methods:**

We analyzed killer cell activating receptor–associated protein expression in colon tissues from patients with ulcerative colitis and established dextran sulfate sodium–induced colitis models in killer cell activating receptor–associated protein knockout mice to determine the potential role of killer cell activating receptor–associated protein in the induction of mucosal inflammation. To clarify the immunoregulation of neutrophils on intestinal epithelial barrier function, we cocultured neutrophils isolated from killer cell activating receptor–associated protein knockout or wild-type mice with intestinal epithelial cells. Additionally, we profiled neutrophil tryptophan metabolites using targeted metabolomics.

**Results:**

Our data demonstrated that killer cell activating receptor–associated protein was highly expressed in the intestinal mucosal tissue and peripheral blood neutrophils of patients with active ulcerative colitis, particularly in neutrophils. Inhibition of killer cell activating receptor–associated protein promoted anti-inflammatory immune responses in neutrophils in vitro, showing a significant decrease in the expression of proinflammatory cytokines, including interleukin6, tumor necrosis factor-α, and interleukin1β. Consistently, killer cell activating receptor–associated protein deficiency alleviated dextran sulfate sodium–induced colitis in mice and improved intestinal mucosal barrier integrity via the aryl hydrocarbon receptor–interleukin22 pathway in neutrophils. Mechanistically, killer cell activating receptor–associated protein deficiency enhanced the nuclear translocation of aryl hydrocarbon receptor through the indoleamine 2,3-dioxygenase 1–kynurenine pathway in neutrophils. Notably, administration of killer cell activating receptor–associated protein knockout neutrophils alleviated mucosal inflammation in dextran sulfate sodium–induced colitis mice.

**Conclusions:**

Overall, our study highlights the critical role of killer cell activating receptor–associated protein in mucosal inflammation in ulcerative colitis by promoting neutrophil proinflammatory immune responses and disrupting the intestinal mucosal barrier integrity, suggesting that targeting killer cell activating receptor–associated protein in neutrophils may represent a novel therapeutic approach for ulcerative colitis.

## Graphical abstract

**Figure:**
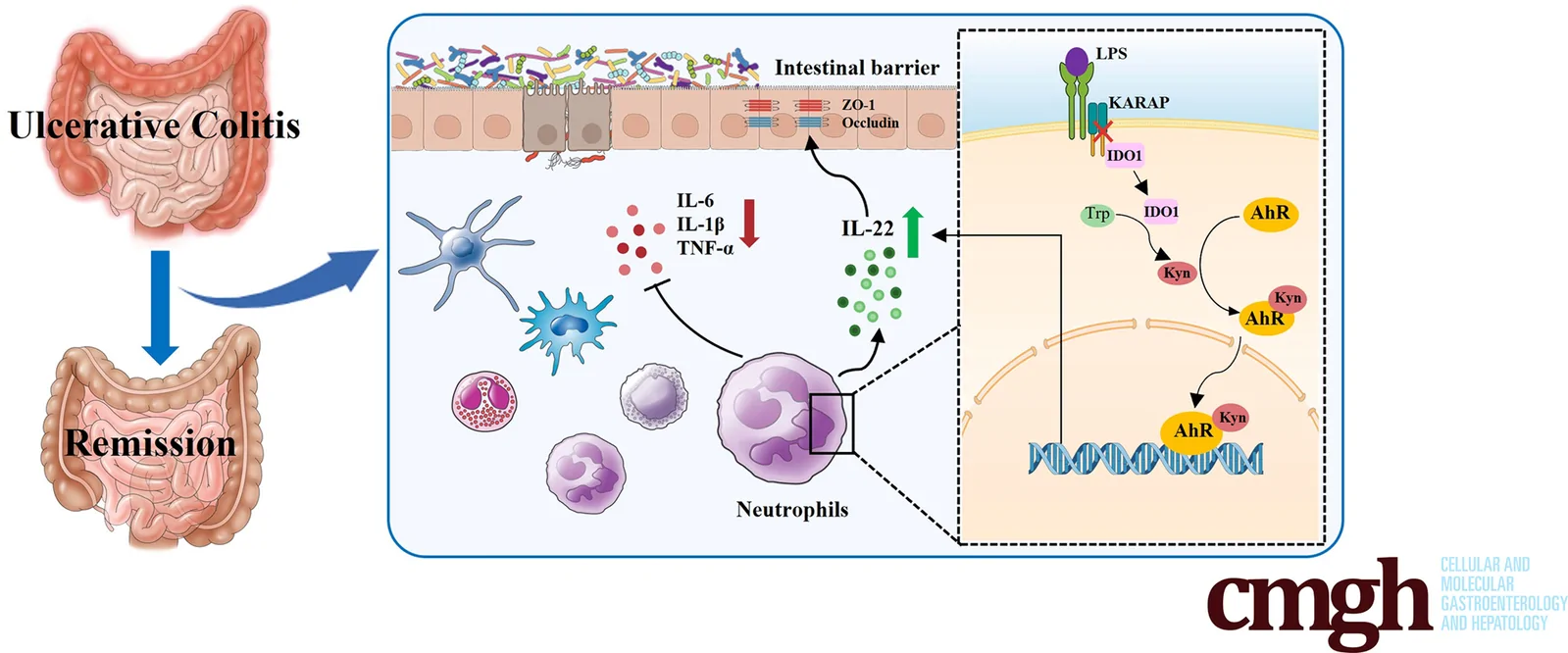

**Summary**

Ulcerative colitis is associated with excessive neutrophil activation and disruption of intestinal barrier integrity. We identify killer cell activating receptor–associated protein as a key regulator of neutrophil-mediated intestinal inflammation and demonstrate that killer cell activating receptor–associated protein deficiency alleviates intestinal mucosal inflammation via the indoleamine 2,3-dioxygenase 1–kynurenine–aryl hydrocarbon receptor–interleukin22 pathway.

**What You Need to Know**

**Background**

Killer cell activating receptor–associated protein plays an important role in regulating autoimmunity and inflammation. However, the molecular mechanisms underlying intestinal mucosal inflammation in ulcerative colitis remain obscure.

**Impact**

Our study reveals that killer cell activating receptor–associated protein exacerbates intestinal mucosal inflammation in ulcerative colitis by promoting proinflammatory immune responses of neutrophils and disrupting intestinal barrier integrity through the indoleamine 2,3-dioxygenase 1–kynurenine-mediated AhR–interleukin22 pathway.

**Future Directions**

Targeting killer cell activating receptor–associated protein in neutrophils may provide a novel therapeutic approach for ulcerative colitis. Further investigation on the potential roles of killer cell activating receptor–associated protein in regulating other cell types and the microbiota in the pathogenesis of ulcerative colitis is warranted.

Ulcerative colitis (UC) is a chronic and relapsing intestinal inflammatory disease caused by an abnormal mucosal immune response.^1^ The pathogenesis of UC is complex and influenced by multiple internal and external factors, including genetic and environmental risks, immune factors, intestinal mucosal barriers, and intestinal microbiota.^2^ When gut inflammation occurs, intestinal epithelial barrier function is compromised, and microbial or food antigens in the intestinal lumen are displaced into the lamina propria of the intestinal mucosa. Innate and acquired immune cells are successively activated and highly infiltrate the intestinal mucosa to elicit a profound immune response and to cause mucosal damage.^3^ The number of neutrophils increases significantly in the intestinal mucosa of patients with UC.^4^

Neutrophils are generally the first leukocytes to be recruited to the site of inflammation, and activated neutrophils perform a variety of antimicrobial functions, such as degranulation, phagocytosis, release of reactive oxygen species and antibacterial proteins, generation of neutrophil extracellular traps, and elimination of intestinal microbial infections.^5^ Moreover, neutrophils promote the migration of other immune cells and mucosal healing by releasing mediators that play key roles in maintaining intestinal epithelial homeostasis.^6^ Tumor necrosis factor-α (TNF-α), interleukin6 (IL6), and interleukin1β (IL1β) are key proinflammatory cytokines that promote the migration and activation of inflammatory cells, exacerbating the inflammatory response. They also increase intestinal permeability, leading to further inflammation and tissue damage.^6^, ^7^, ^8^ Interleukin13 (IL13) significantly affects the functionality of intestinal epithelial cells, resulting in compromised epithelial barrier integrity.^9^^,^^10^ Our previous study reported that CD177^+^ neutrophils produced high levels of interleukin22 (IL22) and further protected the intestinal barrier function from mucosal inflammation.^11^^,^^12^ Therefore, neutrophils play an essential role in maintaining the integrity of the intestinal mucosal barrier by rapidly, efficiently, and precisely eliminating invading pathogens.

Killer cell activating receptor–associated protein (KARAP), also known as TYRO protein tyrosine kinase binding protein, is a member of the type I transmembrane adaptor protein family and is widely expressed on the surface of neutrophils, mononuclear macrophages, natural killer cells, and dendritic cells. As an important adaptor protein, KARAP is reported to be involved in the activation, maturation, and migration of immune cells by sending signals through the immune receptor tyrosine-based activation motif in its cytoplasmic region.^13^ Previous studies have reported that KARAP participates in the occurrence and development of inflammation and autoimmune diseases, such as rheumatoid arthritis, osteoarthritis, and autoimmune encephalomyelitis.^14^ Furthermore, studies have demonstrated that KARAP was highly expressed in colon samples from patients with Crohn’s disease. In the dextran sulfate sodium (DSS) colitis mice model, the absence of KARAP mitigated colon damage, whereas the overexpression of KARAP significantly worsened DSS-induced colon injury.^15^ However, the immunoregulatory role of KARAP and the underlying mechanisms involved in intestinal mucosal inflammation of UC remains undefined.

In our study, we found that KARAP was highly expressed in intestinal mucosal tissue and peripheral blood mononuclear cells of patients with UC, particularly in neutrophils. The inhibition of KARAP promoted anti-inflammatory immune responses in neutrophils. KARAP deficiency improved the intestinal mucosal barrier integrity through the aryl hydrocarbon receptor (AhR)–IL22 pathway and enhanced the nuclear translocation of AhR via indoleamine 2,3-dioxygenase 1 (IDO1)–Kyn (kynurenine) in neutrophils. Notably, KARAP-deficient neutrophils alleviated mucosal inflammation in DSS-induced colitis mice. Therefore, our findings reveal that KARAP plays a vital role in alleviating intestinal mucosal inflammation by improving the intestinal barrier integrity through the downregulation of neutrophil immune response in the IDO1–Kyn–AhR-dependent pathway.

## Results

### Killer Cell Activating Receptor–Associated Protein Is Highly Expressed in Inflamed Colonic Mucosa From Patients With Ulcerative Colitis, Particularly in Neutrophils

KARAP is associated with the occurrence and development of various inflammatory diseases (eg, Alzheimer’s disease, Parkinson’s disease, and various other neurodegenerative diseases).^16^ However, the role of KARAP in intestinal mucosal inflammation remains unclear. To investigate this, we utilized a publicly available RNA sequencing (RNA-seq) dataset of biopsies from patients with UC (GEO: GSE109142), in which patients with UC were classified into mild, moderate, and severe categories based on Mayo score.^17^ Our analysis revealed that KARAP expression was significantly elevated in the colonic mucosa from patients with moderate and severe UC compared with that from patients with mild UC (Figure 1*A*). Moreover, we observed that KARAP expression was positively correlated with the expression of selected signature genes in neutrophil, such as NCF1, CSF3R, S100A8, S100A9, and FCGR3B, in patients with UC (Figure 1*B*), and the expression levels of these signature genes showed a positive correlation with KARAP expression in mild, moderate, and severe disease categories (Figure 1*C*). To explore the role of KARAP in intestinal inflammation of UC, we obtained intestinal mucosal samples from patients with UC and healthy controls (HCs) in our hospital and observed that the levels of KARAP messenger RNA and protein were significantly increased in the inflamed mucosa from patients with UC compared with those from HCs (Figure 1*D* and *E*). Additionally, KARAP expression was also positively correlated with the Ulcerative Colitis Endoscopic Index of Severity, Mayo Score, erythrocyte sedimentation rate, and C-reactive protein, respectively, in patients with active UC (data not shown).

**Figure 1:**
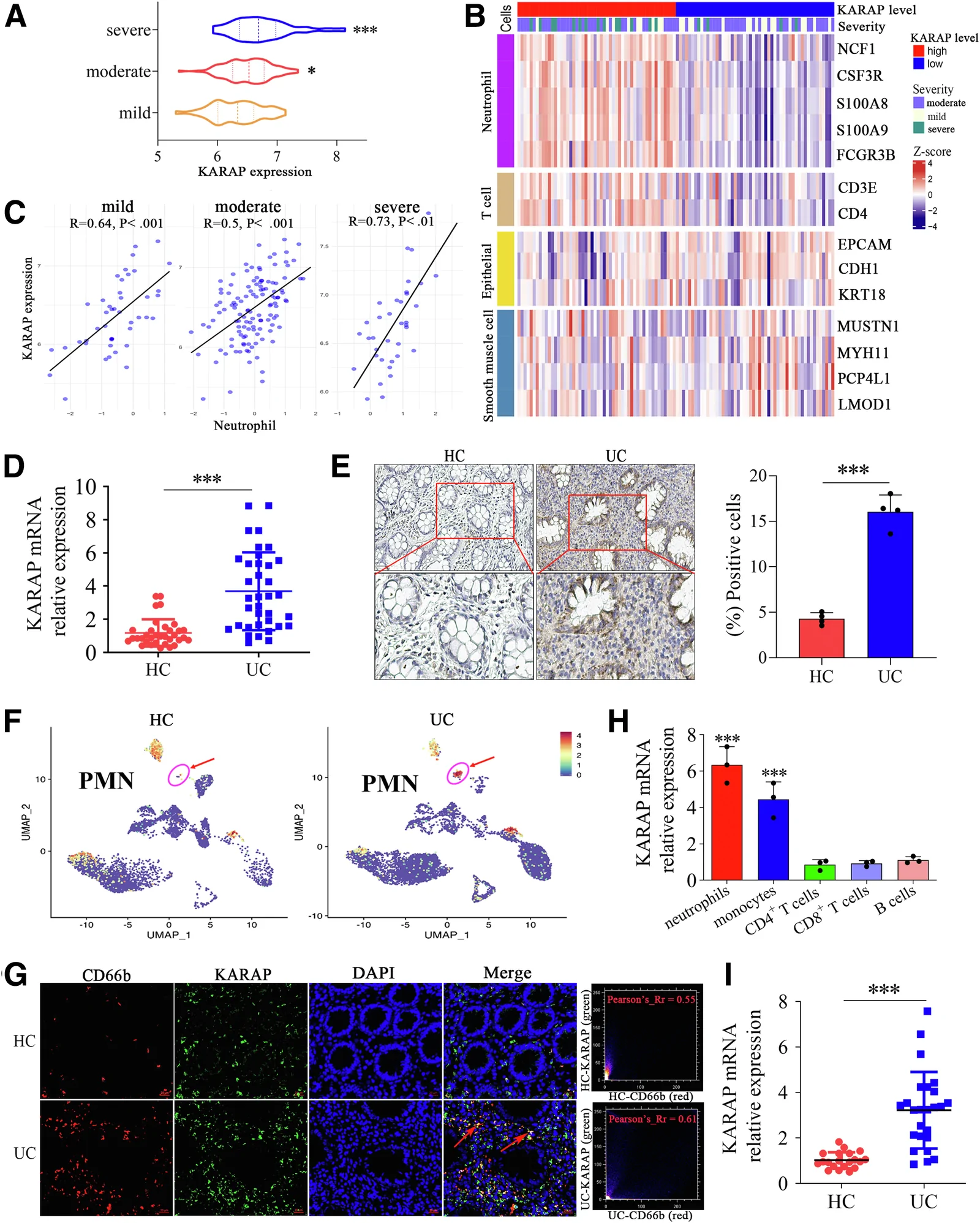
**KARAP is highly expressed in inflamed colonic mucosa from patients with UC, particularly in neutrophils.** (*A*) Bioinformatics analysis of RNA-seq data from rectal biopsies of patients with UC. KARAP expression was analyzed in patients with disease severity. (*B*) Heatmap of signature genes in patients with UC with high vs low KARAP expression. (*C*) Correlation analysis between neutrophil signature genes and expression of KARAP in biopsies from patients with UC. (*D*) qRT-PCR analysis of KARAP messenger RNA (mRNA) levels in intestinal mucosal tissues from patients with UC (n = 36) and HCs (n = 34). (*E*) Representative immunohistochemistry staining of KARAP in intestinal mucosa from patients with UC and HCs. Scale bar, 50 μm. (*F*) Analysis of KARAP expression in neutrophils from intestinal mucosa of patients with UC and HCs by single-cell RNA-seq (scRNA-seq). (*G*) Immunofluorescence staining KARAP and CD66b in intestinal mucosa. Scale bar, 20 μm. (*H*) qRT-PCR analysis of KARAP expression in immune cell subsets. ∗∗∗*P* < .001 vs CD8^+^ T cells. (*I*) qRT-PCR analysis of KARAP mRNA levels in peripheral blood neutrophils from patients with UC (n = 20) and HCs (n = 25). ∗*P* < .05, ∗∗∗*P* < .001. DAPI, 4′,6-diamidino-2-phenylindole; UMAP, Uniform Manifold Approximation and Projection.

Given the central role of immune cells in intestinal mucosal inflammation,^18^^,^^19^ we performed single-cell RNA-seq on intestinal mucosal samples from patients with active UC and HCs to determine the phenotypic expression of KARAP in different immune cells. Our analysis revealed high KARAP expression in neutrophils of intestinal mucosa from both patients with UC and HC, which was markedly increased in neutrophils from patients with active UC (Figure 1*F*). We further validated this finding using fluorescence costaining of KARAP and CD66b and observed a substantial increase of KARAP in CD66b^+^ neutrophils in the inflamed mucosa of patients with UC (Figure 1*G*). To phenotypically analyze the expression of KARAP in different immune cells, we isolated different immune cells from peripheral blood and found that KARAP was highly expressed in neutrophils and CD14^+^ monocytes from HCs (Figure 1*H*) and was significantly upregulated in peripheral blood neutrophils from patients with UC compared with HCs (Figure 1*I*). Therefore, these data demonstrate that KARAP is highly expressed in neutrophils and may play a critical role in the pathogenesis of UC.

### Blockage of Killer Cell Activating Receptor–Associated Protein Promotes Anti-inflammatory Immune Responses in Neutrophils

As one of crucial components of the innate immune system, neutrophils play a critical role in combating invading pathogens and regulating inflammatory responses.^20^ To investigate the immunomodulatory role of KARAP in regulating neutrophil functions, we isolated neutrophils from the bone marrow of wild-type (WT) and KARAP knockout (KARAP^−/−^) mice. Similarly, the expression of proinflammatory cytokines (eg, IL6, TNF-α, and IL1β) was significantly decreased in KARAP^−/−^ neutrophils compared with WT neutrophils (Figure 2*A*). Furthermore, KARAP deficiency attenuated the phosphorylation of key proteins (including P65, P38, Erk, AKT, and mTOR) in several inflammatory signaling pathways (data not shown). Next, we cocultured neutrophils with fluorescein isothiocyanate (FITC)-labeled *Escherichia coli* for 3 hours and analyzed FITC fluorescence intensity by flow cytometry to evaluate the phagocytosis. The phagocytic capacity of KARAP^−/−^ neutrophils was markedly enhanced compared with that of WT neutrophils (Figure 2*B*). To assess bactericidal activity, neutrophils were cocultured with *E coli* for 3 hours. The coculture supernatants were plated on Luria-Bertani agar and incubated overnight. The number of colony-forming units was then quantified. Colony-forming units from KARAP^−/−^ neutrophil cocultures were significantly decreased, indicating an increase in the bactericidal capacity of KARAP^−/−^ neutrophils (Figure 2*C*). Moreover, ImageJ analysis of immunofluorescence images revealed that citrullinated histone 3 expression was increased in KARAP^−/−^ neutrophils compared with WT controls (Figure 2*D*). The same pattern of citrullinated histone 3 expression was seen in KARAP^−/−^ neutrophils by Western blot analysis (Figure 2*E*). Taken together, these results indicate that an inhibition of KARAP expression could promote the anti-inflammatory and antimicrobial functions of neutrophils.

**Figure 2:**
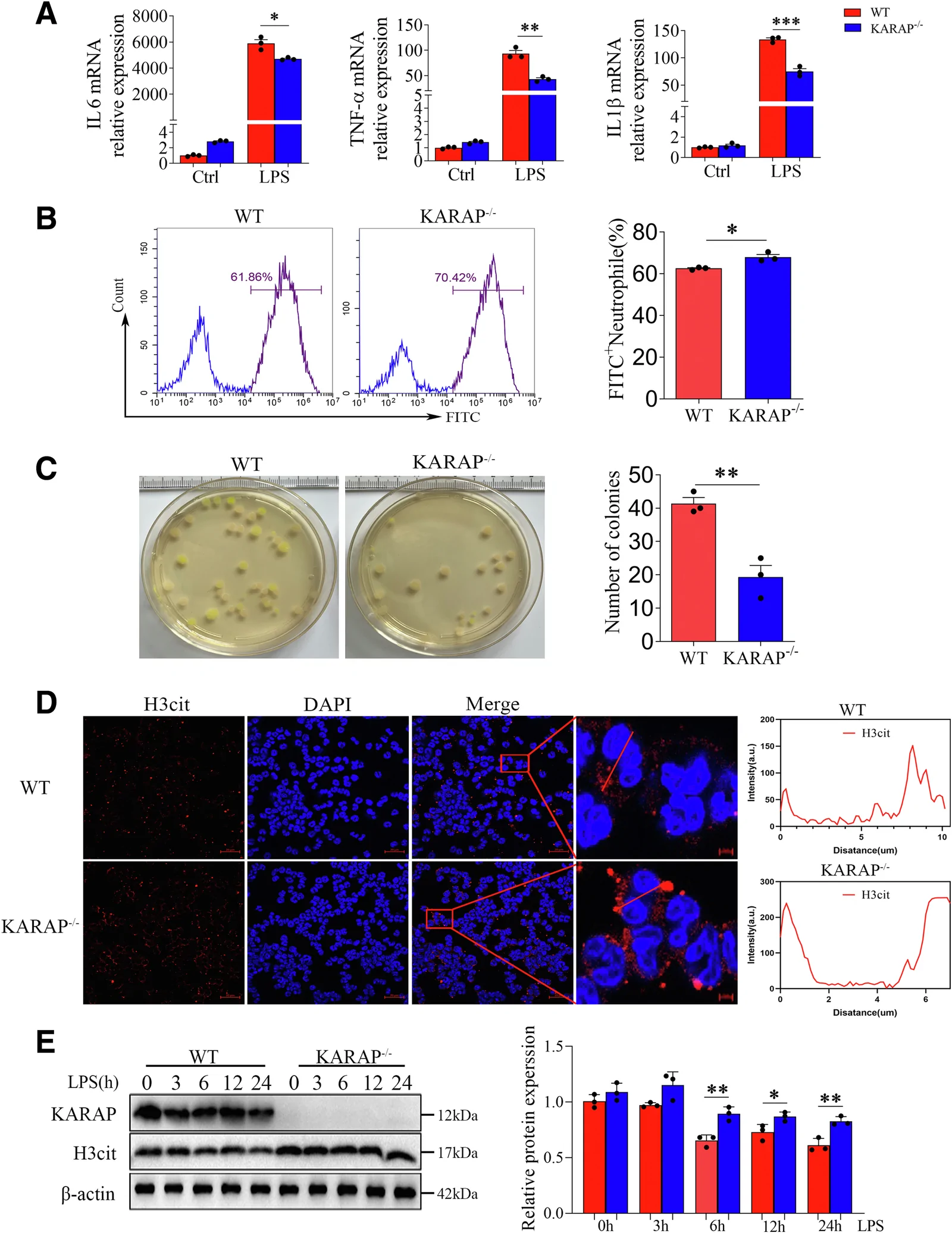
**KARAP inhibition promotes an anti-inflammatory responsein neutrophils.** (*A*) Neutrophils were isolated from the bone marrow of WT and KARAP^−/−^ mice, and the expression of IL6, TNF-α, and IL1β was analyzed by qRT-PCR. (*B*) Phagocytic function of bone marrow-derived neutrophils from WT and KARAP^−/−^ mice was assessed by flow cytometry after coculture with FITC-labeled *E coli*. (*C*) Phagocytic function of bone marrow-derived neutrophils from WT and KARAP^−/−^ mice was assessed by colony-forming units assay of the coculture supernatant. (*D*) Immunofluorescence staining and quantification of citrullinated histone 3 (H3cit) in LPS-treated (200 ng/mL) neutrophils. Scale bar, 20 μm. (*E*) Western blot analysis of H3cit protein expression in neutrophils treated with LPS for the indicated durations. ∗*P* < .05, ∗∗*P* < .01, ∗∗∗*P* < .001. DAPI, 4′,6-diamidino-2-phenylindole.

### Killer Cell Activating Receptor–Associated Protein Deficiency Ameliorates Intestinal Mucosal Inflammation in Mice

To further investigate the role of KARAP in intestinal mucosal inflammation, we established a DSS-induced colitis model in WT and KARAP^−/−^ mice. As shown in Figure 3*A*, KARAP^−/−^ mice exhibited significantly less weight loss than WT mice during the 10-day observation period. All mice were sacrificed on day 10, showing that colon edema and thickening were significantly relieved and that colon length was significantly improved in KARAP^−/−^ mice compared with those in WT mice (Figure 3*B*). Pathologic analysis further showed that the infiltration of inflammatory cells in the colon of KARAP^−/−^ mice was significantly reduced and that pathologic scores were lower in KARAP^−/−^ mice than those of WT mice (Figure 3*C*). Colon tissues were collected from DSS-induced colitis mice to measure the levels of inflammatory cytokines. The expression of IL6, IL1β, and TNF-α was markedly lower in colon tissues from KARAP^−/−^ mice than those from WT mice (Figure 3*D*). We measured the serum levels of inflammatory cytokines in DSS-induced colitis mice by enzyme-linked immunosorbent assay and found that the levels of IL6, IL1β, and TNF-α were markedly decreased in sera from KARAP^−/−^ mice compared with those from WT mice (Figure 3*E*). To assess intestinal barrier function in WT and KARAP^−/−^ mice after DSS exposure, mice were fasted for 12 hours and then orally gavaged with FITC-dextran prior to dissection. After 4 hours, the distribution of FITC-dextran in vivo was visualized using a small animal imaging system. We found that KARAP^−/−^ mice exhibited less FITC-dextran leakage in the intestinal mucosa compared with WT controls (Figure 4*A*). Consistent with this, serum levels of FITC-dextran were significantly lower in KARAP^−/−^ mice than in WT mice (Figure 4*B*). Analysis of tight junction proteins in intestinal mucosal tissues revealed that the expression of Zonula Occludens-1 (ZO-1) and Occludin was significantly increased in DSS-treated KARAP^−/−^ mice compared with WT controls (Figure 4*C* and *D*). Therefore, these data indicate that KARAP inhibition ameliorates mucosal inflammation and relieves the intestinal mucosal barrier damage in DSS-induced colitis mice.

**Figure 3:**
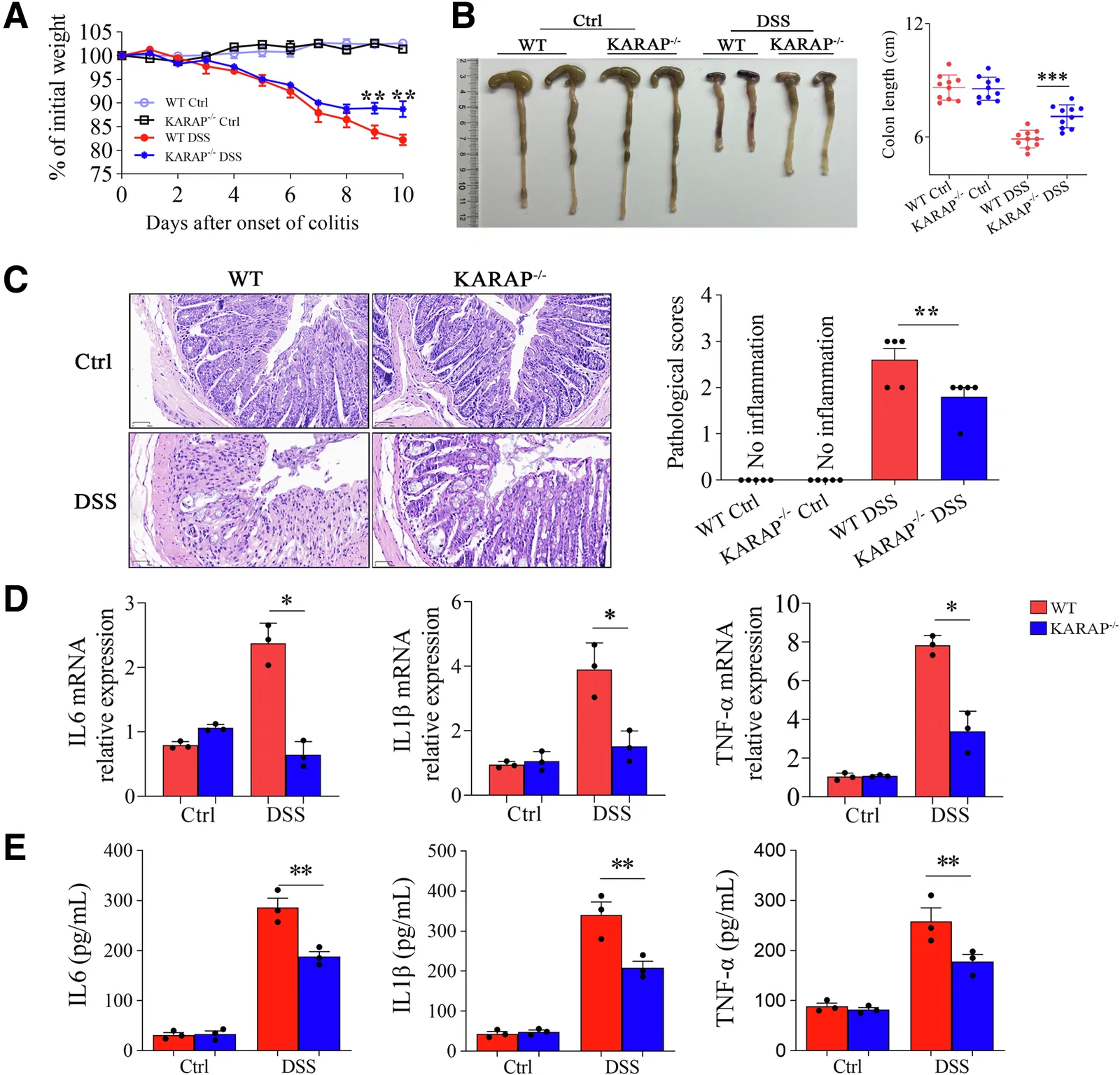
**KARAP deficiency ameliorates DSS-induced colitis in mice.** (*A*) Body weight changes shown as percentage of initial weight. ∗∗*P* < .01, DSS-treated KARAP^−/−^ vs DSS-treated WT mice at the same timepoint. (*B*) The length of colons is shown. (*C*) Representative hematoxylin and eosin–stained colon sections and corresponding histopathologic scores. Scale bar, 50 μm. (*D*) qRT-PCR analysis of proinflammatory cytokine messenger RNA (mRNA) levels in colonic mucosa. (*E*) Peripheral blood serum was collected from WT and KARAP^−/−^ mice with or without DSS treatment. Concentrations of IL6, IL1β, and TNF-α were measured by enzyme-linked immunosorbent assay. Data are presented as mean ± standard error of the mean. ∗*P* < .05, ∗∗*P* < .01, ∗∗∗*P* < .001.

**Figure 4:**
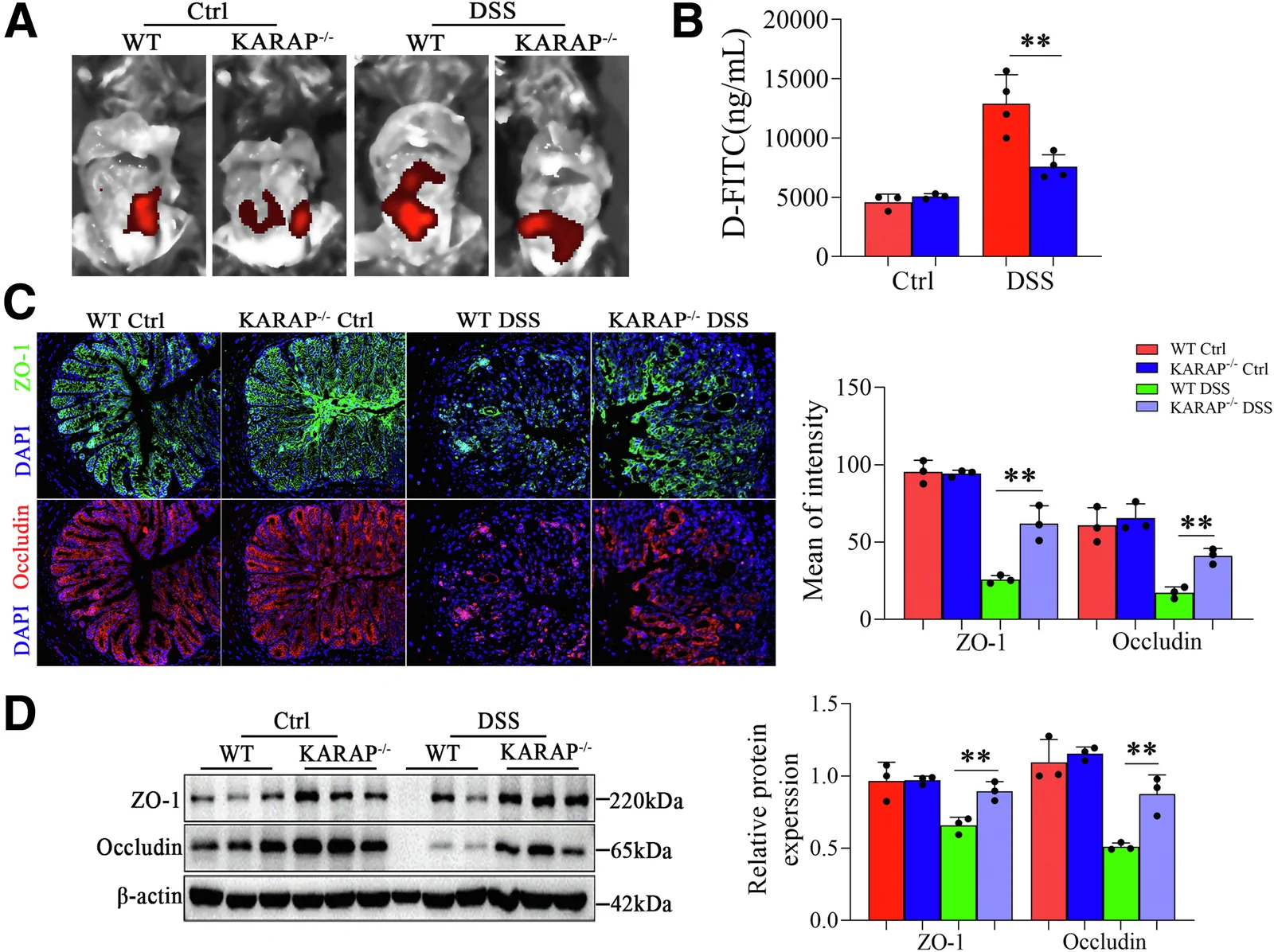
**KARAP deficiency alleviates intestinal barrier integrity damage.** (*A*) In vivo imaging of mice 4 hours after oral gavage with FITC-dextran (50 mg/kg). (*B*) Serum concentration of FITC-dextran measured by fluorometry. (*C*) Immunofluorescence staining of tight junction proteins ZO-1 and Occludin in colonic mucosa. Scale bar, 50 μm. (*D*) Western blot analysis of ZO-1 and Occludin protein expression in colonic mucosa. ∗∗*P* < .01. DAPI, 4′,6-diamidino-2-phenylindole.

### Killer Cell Activating Receptor–Associated Protein Deficiency Promotes Intestinal Mucosal Barrier Integrity via Upregulation of Aryl Hydrocarbon Receptor in Neutrophils

To further investigate the role of neutrophils in intestinal epithelial barrier function after KARAP deficiency, we established a neutrophil-Caco-2 cell coculture model. We found the transepithelial resistance (TEER) was significantly higher in Caco-2 cells cocultured with KARAP^−/−^ neutrophils compared with those with WT neutrophils (Figure 5*A*). FITC-dextran permeability assays revealed that the permeability of Caco-2 cells cocultured with KARAP^−/−^ neutrophils was significantly lower than that of those with WT neutrophils (Figure 5*B*). Furthermore, we assessed the expression of tight junction proteins in Caco-2 cells cocultured with either WT or KARAP^−/−^ neutrophils and found ZO-1 expression was significantly higher in Caco-2 cells cocultured with KARAP^−/−^ neutrophils than that with WT neutrophils (Figure 5*C–E*). These data suggest that KARAP deficiency in neutrophils promotes intestinal mucosal barrier integrity.

**Figure 5:**
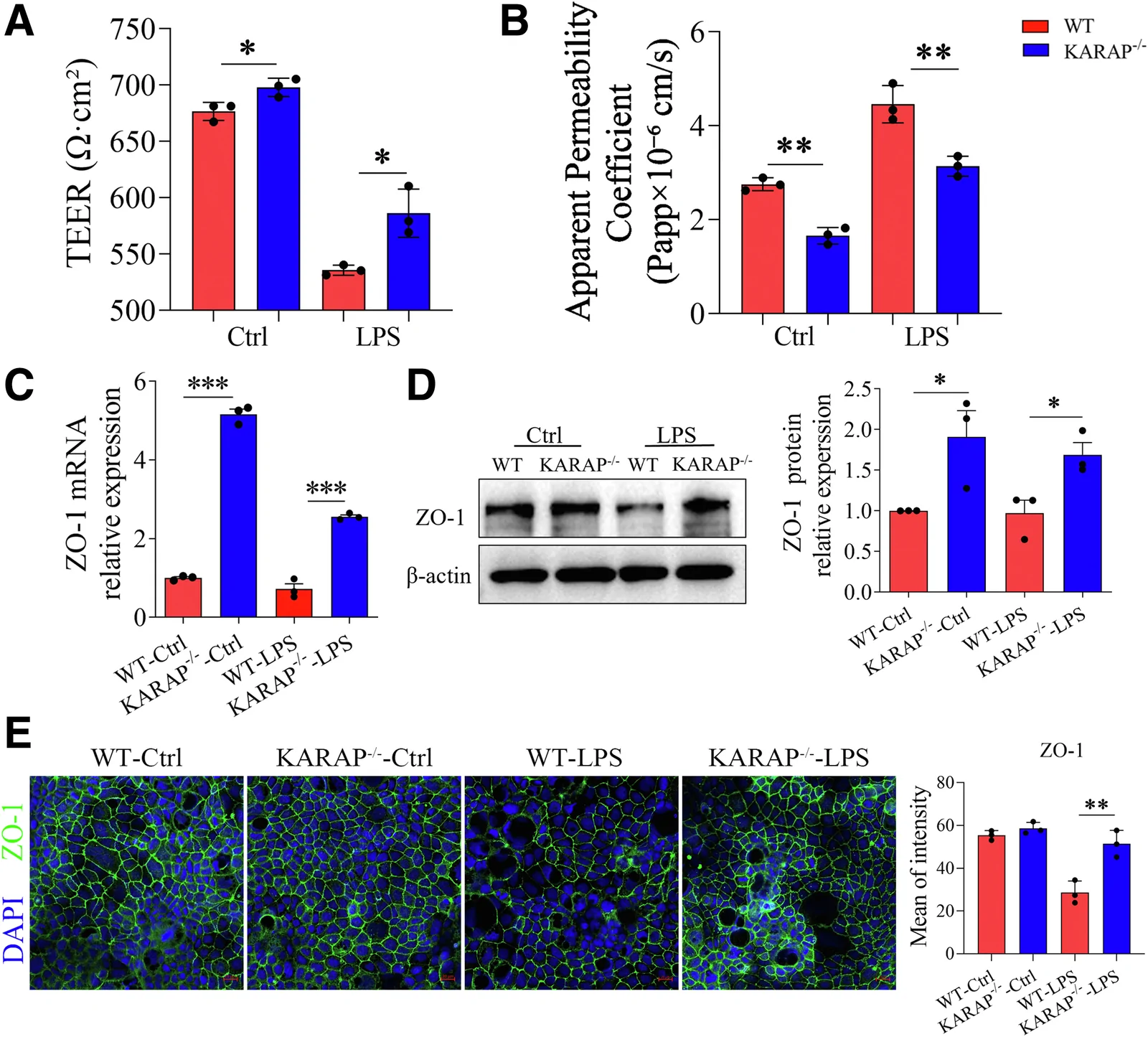
**KARAP deficiency in neutrophils improves the intestinal barrier integrity.** Neutrophils (1 × 10^6^) were isolated from the bone marrow of WT and KARAP^−/−^ mice, and cocultured with Caco-2 cells (2 × 10^5^) for 24 hours. (*A* and *B*) The TEER and permeability of Caco-2 cells were analyzed. (*C* and *D*) Analysis of ZO-1 expression by qRT-PCR and Western blot. (*E*) Immunofluorescence analysis of ZO-1 expression in Caco-2 cells after coculture with neutrophils. Scale bar, 20 μm. ∗*P* < .05, ∗∗*P* < .01, ∗∗∗*P* < .001. DAPI, 4′,6-diamidino-2-phenylindole; mRNA, messenger RNA.

To further investigate the role of KARAP in regulating neutrophil functions, we isolated neutrophils from the bone marrow of WT and KARAP^−/−^ mice for RNA-seq analysis. We found that there were 789 differentially expressed genes between WT and KARAP^−/−^ neutrophils in the DSS model and that AhR expression was significantly higher in KARAP^−/−^ neutrophils than in WT neutrophils (Figure 6*A*). AhR expression was identified by isolating the nuclear and cytoplasmic proteins of neutrophils, and increased levels of AhR were observed in both nucleus and cytoplasm of KARAP^−/−^ neutrophils compared with WT controls (Figure 6*B*). Immunofluorescence analysis revealed enhanced nuclear localization of AhR in KARAP^−/−^ neutrophils compared with WT controls (Figure 6*C*; Supplementary Videos). Because previous studies have reported that AhR activation repairs the damaged intestinal barrier,^20^ we treated neutrophils with the AhR activator 6-formylindolo[3,2-b]carbazole (FICZ) or the inhibitor CH223191 and established a neutrophil–Caco-2 cell coculture model to investigate the role of AhR in regulating intestinal barrier integrity. We found the TEER was significantly higher in Caco-2 cells cocultured with KARAP^−/−^ neutrophils compared with those with WT neutrophils, and this enhancement was abolished when KARAP^−/−^ neutrophils were treated with CH223191; the addition of FICZ to Caco-2 cells cocultured with WT neutrophils resulted in an increased TEER compared with the control group without the addition (Figure 6*D*). Concurrently, FITC-dextran permeability assays revealed that the permeability of Caco-2 cells cocultured with KARAP^−/−^ neutrophils was significantly lower than that of those with WT neutrophils, this reduction was negated by the treatment of KARAP^−/−^ neutrophils with CH223191, and the addition of FICZ to Caco-2 cells cocultured with WT neutrophils led to a decrease in FITC-dextran permeability compared with the control group that did not receive this addition (Figure 6*E*). Furthermore, we assessed tight junction proteins expression in Caco-2 cells cocultured with either WT or KARAP^−/−^ neutrophils and found the enhancement of ZO-1 and Occludin expression was abolished when KARAP^−/−^ neutrophils were treated with CH223191. In contrast, CH223191 had no effect in cocultures with WT neutrophils (Figure 6*F* and *G*). The immunofluorescence detection of ZO-1 expression corroborated these findings (Figure 6*H*). Collectively, these data indicate that KARAP deficiency improves intestinal barrier integrity through the AhR pathway in neutrophils.

**Figure 6:**
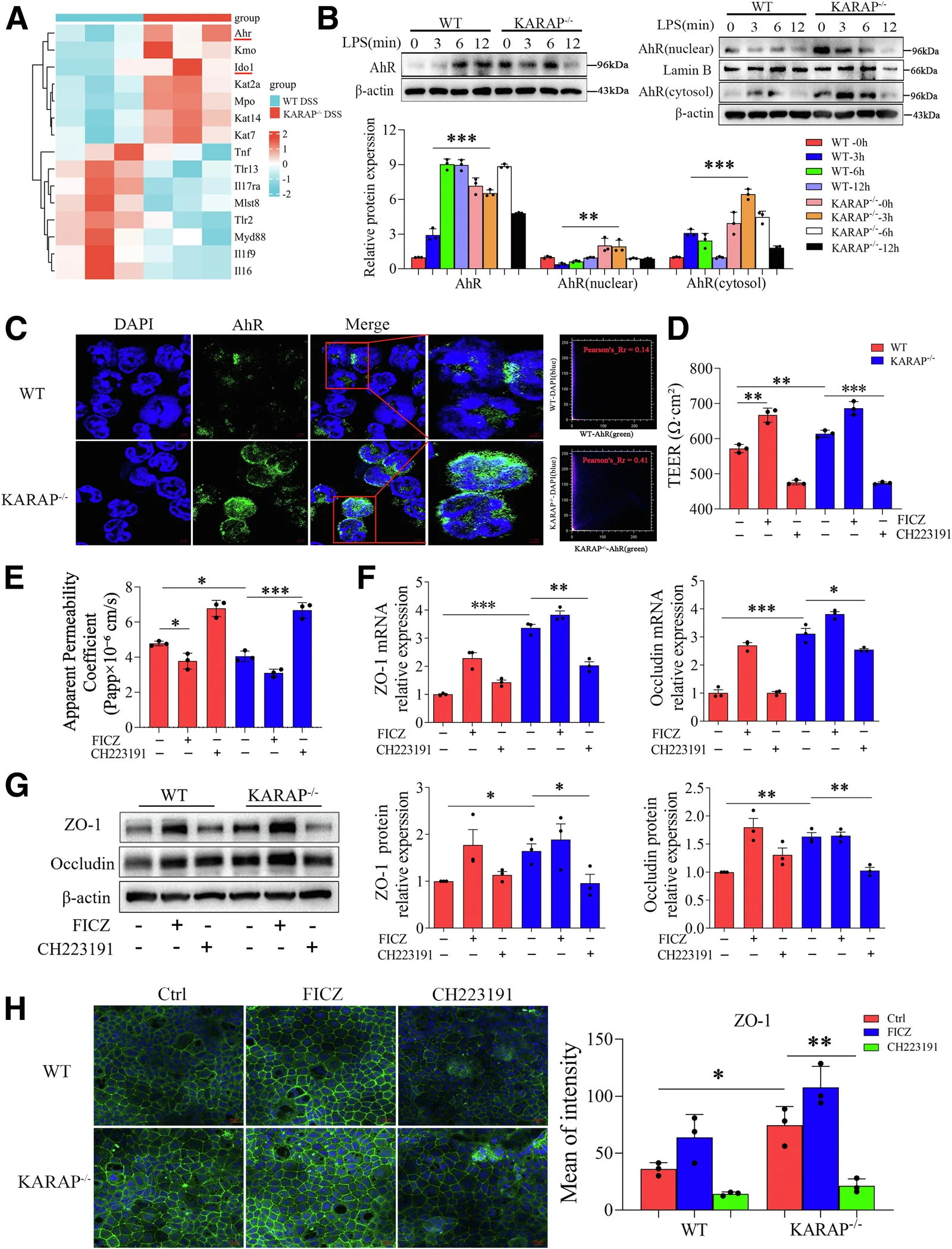
**KARAP deficiency improves the intestinal barrier integrity via the AhR pathway in neutrophils.** (*A*) Heatmap of differentially expressed genes in bone marrow neutrophils from DSS-treated WT and KARAP^−/−^ mice (RNA-seq data). (*B*) Western blot analysis of AhR protein levels in whole cell, cytoplasmic, and nuclear fractions of LPS-stimulated (200 ng/mL) neutrophils. (*C*) Immunofluorescence staining of AhR in LPS-stimulated neutrophils. Scale bar, 20 μm. (*D* and *E*) Neutrophils were isolated from the bone marrow of WT and KARAP^−/−^ mice, and cocultured with Caco-2 cells for 24 hours with the agonist FICZ (1 μM) or inhibitor CH223191 (10 μM). The TEER and permeability of Caco-2 cells were analyzed. (*F* and *G*) qRT-PCR and Western blot analysis of ZO-1 and Occludin expression in neutrophils treated with the agonist FICZ (1 μM) or inhibitor CH223191 (10 μM). (*H*) Immunofluorescence analysis of ZO-1 expression in Caco-2 cells after coculture with neutrophils. Scale bar, 20 μm. ∗*P* < .05, ∗∗*P* < .01, ∗∗∗*P* < .001. DAPI, 4′,6-diamidino-2-phenylindole; mRNA, messenger RNA.

To further explore the role of AhR in the intestinal mucosal barrier of KARAP^−/−^ mice, we established a DSS-induced colitis model in WT and KARAP^−/−^ mice and treated them with simultaneous intraperitoneal injection of FICZ or CH223191. We found that CH223191-treated KARAP^−/−^ mice exhibited significantly more weight loss, shortened colon length, and greater inflammatory cell infiltration than their untreated counterparts (Figure 7*A–C*). Furthermore, CH223191 treatment significantly accelerated FITC-dextran leakage in KARAP^−/−^ mice after DSS insult compared with untreated mice (Figure 7*D*). Serum FITC-dextran levels were significantly increased in KARAP^−/−^ mice after intraperitoneal injection of CH223191 (Figure 7*E*). Moreover, ZO-1 expression was significantly lower in CH223191-treated KARAP^−/−^ mice than in untreated KARAP^−/−^ DSS mice (Figure 7*F*). Taken together, these data demonstrate that KARAP deficiency improves the intestinal mucosal barrier integrity in an AhR-dependent pathway in neutrophils.

**Figure 7:**
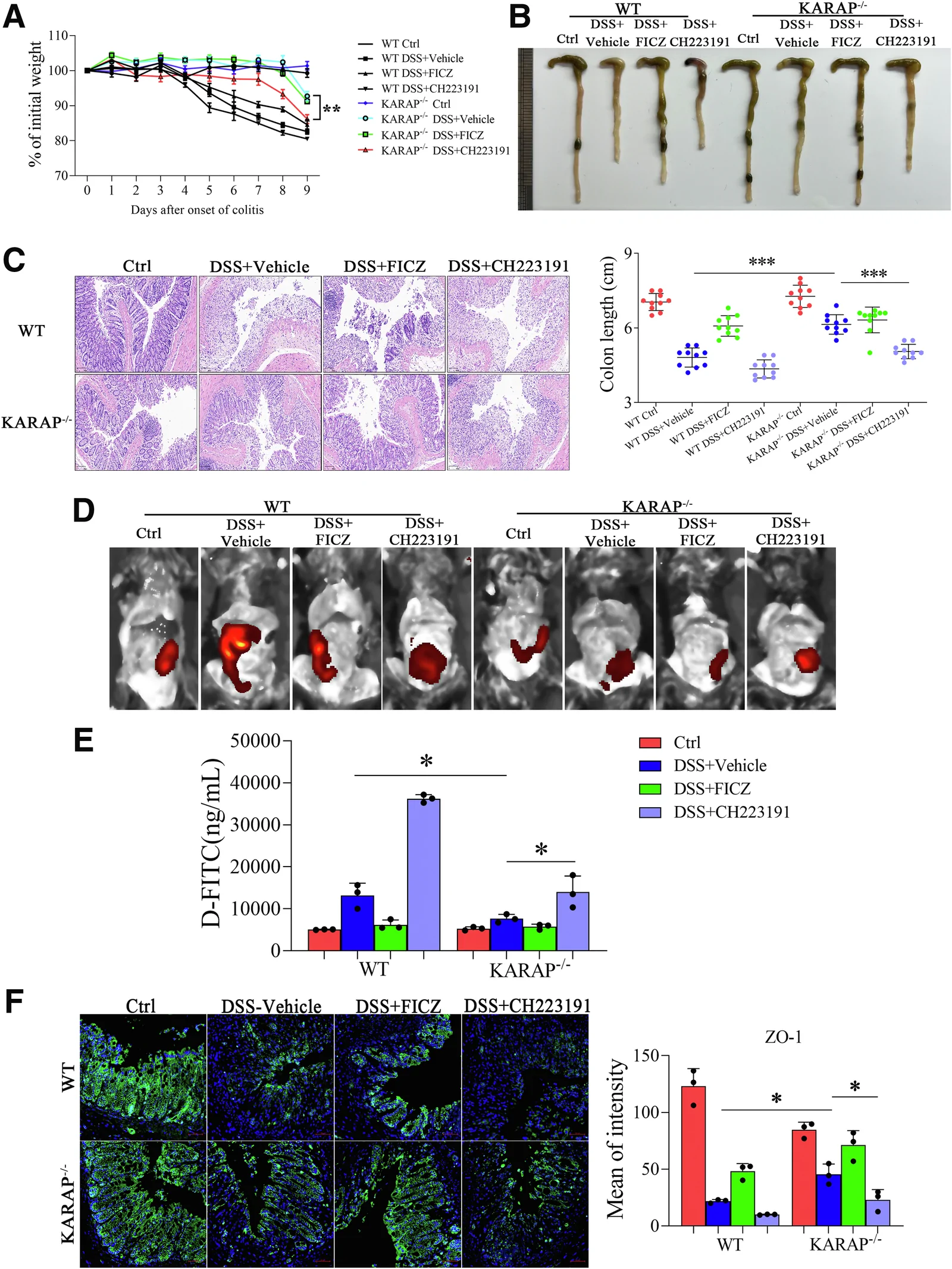
**KARAP deficiency improves the intestinal injury via the AhR pathway in neutrophils.** WT and KARAP^−/−^ mice were administered 2.5% DSS in drinking water to induce colitis. Mice were subsequently treated intraperitoneally with the AhR agonist FICZ or the inhibitor CH223191. Experimental groups included: non-DSS controls (WT Ctrl, KARAP^−/−^ Ctrl), DSS-treated groups (WT DSS + vehicle, KARAP^−/−^ DSS + vehicle), and DSS-treated groups with AhR modulator (WT DSS + FICZ, KARAP^−/−^ DSS + FICZ, WT DSS + CH223191, KARAP^−/−^ DSS + CH223191). (*A*) Body weight changes expressed as a percentage of initial weight over a 9-day period. ∗∗ *P* < .01, KARAP^−/−^ DSS + vehicle mice vs KARAP^−/−^ DSS + CH223191 mice with at the same time points. (*B*) Colon length at sacrifice. (*C*) Representative hematoxylin and eosin–stained colon sections. Scale bar, 50 μm. (*D*) In vivo permeability was assessed by oral gavage of FITC-dextran (50 mg/kg). Representative fluorescence images show FITC-dextran distribution in the gastrointestinal tract 4 hours postgavage. (*E*) Quantification of serum FITC-dextran levels. ∗*P* < .05. (*F*) Representative immunofluorescence images of colon sections stained for the tight junction protein ZO-1. Nuclei are counterstained with 4′,6-diamidino-2-phenylindole (DAPI). Scale bar, 50 μm. ∗*P* < .05, ∗∗*P* < .01, ∗∗∗*P* < .001.

### Killer Cell Activating Receptor–Associated Protein Deficiency in Neutrophils Improves Intestinal Mucosal Barrier Integrity in the Aryl Hydrocarbon Receptor–Interleukin22 Axis–Dependent Manner

Previous studies have shown that AhR regulates IL22 production, which in turn modulates intestinal barrier integrity during mucosal inflammation.^21^ We hypothesized that whether KARAP regulates intestinal mucosal barrier functions, relying on the AhR–IL22 pathway in neutrophils. To test this, we isolated neutrophils from the bone marrow of WT and KARAP^−/−^ mice. Upon lipopolysaccharide (LPS) stimulation, IL22 expression was markedly higher in KARAP^−/−^ neutrophils than in WT mice (Figure 8*A–C*). To investigate the underlying mechanism involved in the immunoregulation of KARAP^−/−^ neutrophils, we cultured KARAP^−/−^ neutrophils under stimulation with CH223191 and found that IL22 was significantly inhibited. On the contrary, addition of FICZ markedly increased IL22 expression in WT neutrophils (Figure 8*D*). To determine whether this effect was mediated by IL22, we conducted neutralization and supplementation experiments. Our results indicated that the TEER was significantly higher in Caco-2 cells cocultured with KARAP^−/−^ neutrophils than that with WT neutrophils, and this enhancement was completely abolished when KARAP^−/−^ neutrophils were treated with an anti-IL22 monoclonal antibody (Figure 8*E*). Furthermore, FITC-dextran permeability assays demonstrated that the permeability of Caco-2 cells cocultured with KARAP^−/−^ neutrophils was significantly lower than that of those cocultured with WT neutrophils, and this reduction in permeability was also negated by the treatment of KARAP^−/−^ neutrophils with the anti-IL22 monoclonal antibody; furthermore, the addition of IL22 to Caco-2 cells co-cultured with WT neutrophils resulted in reduced FITC-dextran permeability compared with the control group that did not receive this addition. (Figure 8*F*). Notably, an inclusion of anti-IL22 monoclonal antibody significantly reduced ZO-1 and Occludin expression in Caco-2 cells co-cultured with KARAP^−/−^ neutrophils but had no significant effect on those cocultured with WT neutrophils by quantitative polymerase chain reaction and Western blot (Figure 8*G* and *H*). Additionally, immunofluorescence assays confirmed that the addition of the anti-IL22 monoclonal antibody significantly reduced ZO-1 expression in Caco-2 cells cocultured with KARAP^−/−^ neutrophils (Figure 8*I*). Therefore, these results demonstrate that KARAP deficiency in neutrophils improves the intestinal mucosal barrier integrity through the AhR–IL22 pathway.

**Figure 8:**
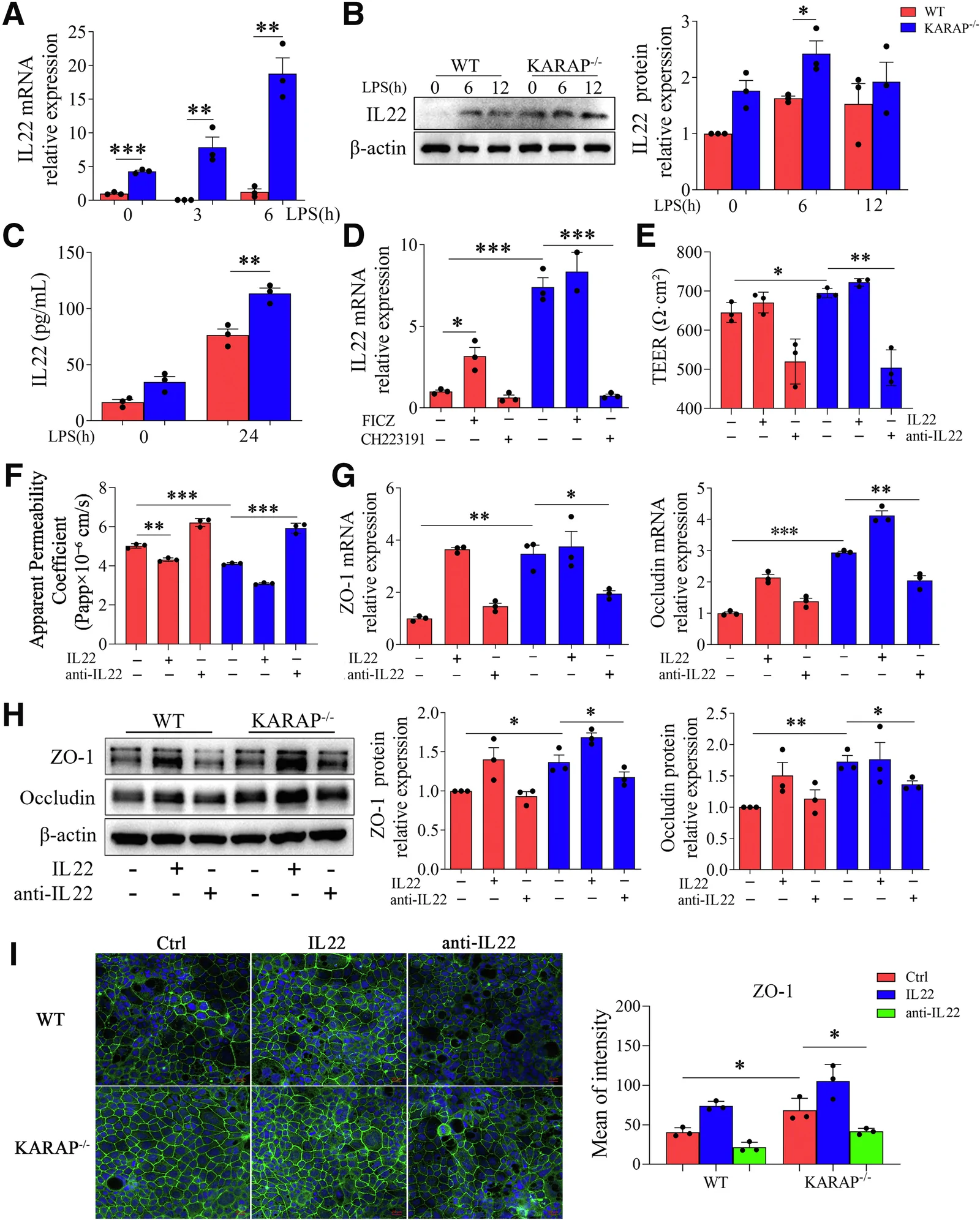
**KARAP deficiency in neutrophils improves the intestinal barrier integrity via the AhR–IL22 axis.** (*A*) qRT-PCR analysis of IL22 messenger RNA (mRNA) expression in neutrophils. (*B* and *C*) Western blot and enzyme-linked immunosorbent assay analysis of IL22 protein expression in neutrophils. (*D*) qRT-PCR analysis of IL22 mRNA in neutrophils treated with FICZ (1 μM) or CH223191 (10 μM). (*E* and *F*) Neutrophils were isolated from the bone marrow of WT and KARAP^−/−^ mice, and cocultured with Caco-2 cells for 24 hours with the recombinant IL22 (10 ng/mL) or neutralizing anti-IL22 antibody (1 μg/mL). The TEER and permeability of Caco-2 cells were analyzed. (*G* and *H*) qRT-PCR and Western blot analysis of ZO-1 and Occludin expression in neutrophils treated with the recombinant IL22 or neutralizing anti-IL22 antibody. (*I*) Immunofluorescence analysis of ZO-1 expression in Caco-2 cells after coculture with neutrophils. Scale bar, 20 μm. ∗*P* < .05, ∗∗*P* < .01, ∗∗∗*P* < .001.

### Killer Cell Activating Receptor–Associated Protein Deficiency in Neutrophils Enhances Nuclear Translocation of Aryl Hydrocarbon Receptor Through an Indoleamine 2,3-Dioxygenase 1–Kynurenine-Dependent Manner

To elucidate how KARAP regulates intestinal mucosal barrier integrity, we performed Kyoto Encyclopedia of Genes and Genomes (KEGG) enrichment analysis on differentially expressed genes from WT and KARAP^−/−^ neutrophils. This analysis identified tryptophan metabolism as a significantly enriched pathway (Figure 9*A*). We therefore conducted targeted metabolomics profiling. After quality control (Figure 9*B*), orthogonal partial least squares-discriminant analysis demonstrated clear separation of tryptophan metabolic profiles between WT and KARAP^−/−^ neutrophils (Figure 9*C*). Targeted metabolomic quantification was then performed. Both hierarchical clustering and Z-score analyses confirmed significantly elevated Kyn levels in KARAP^−/−^ neutrophils (Figures 9*D–F*). KEGG analysis further supported the involvement of the Kyn-associated tryptophan metabolism pathway (Figure 9*G*). As Kyn is an AhR ligand that facilitates its nuclear translocation,^22^ we detected Kyn levels in neutrophils. We found that Kyn levels were significantly higher in KARAP^−/−^ neutrophils than those in WT neutrophils (Figure 9*H*). Previous research has confirmed that IDO1 promotes the metabolism of tryptophan into Kyn.^23^ RNA-seq revealed that IDO1 was upregulated in KARAP^−/−^ neutrophils (Figure 6*A*), and coimmunoprecipitation confirmed its direct physical interaction with KARAP (Figure 9*I*). To elucidate how IDO1 regulates AhR, we then assessed the nuclear translocation of AhR upon IDO1 inhibition using Epacadostat. We found that Epacadostat-mediated inhibition of IDO1 suppressed AhR nuclear translocation. This effect was particularly pronounced in KARAP^−/−^ neutrophils. (Figure 9*J*). Collectively, KARAP deficiency enhances the nuclear translocation of AhR in neutrophils, which was dependent on the IDO1-Kyn pathway.

**Figure 9:**
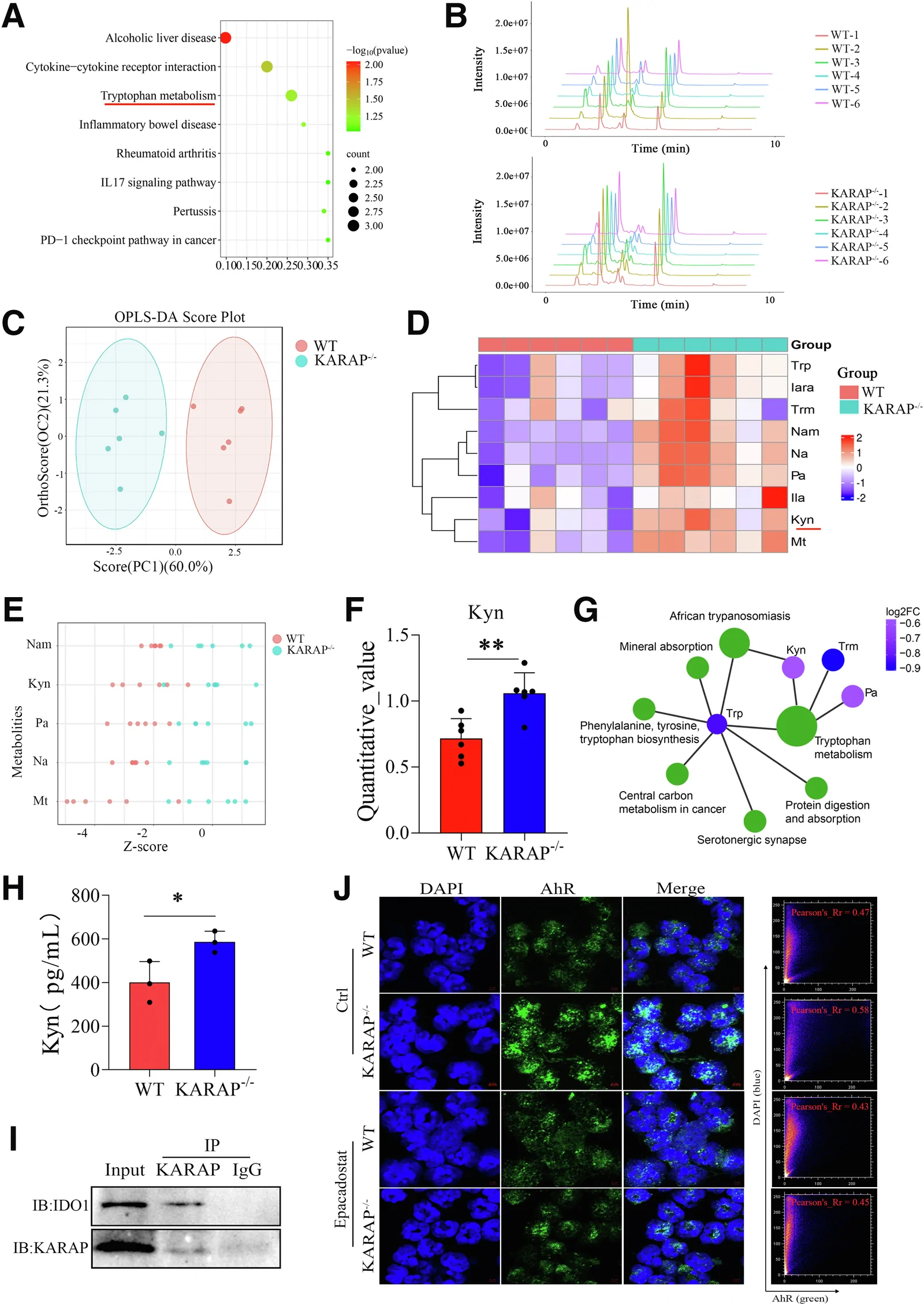
**KARAP deficiency in neutrophils enhances AhR nuclear translocation via the IDO1–Kyn-dependent manner.** (*A*) RNA-seq analysis was performed in neutrophils from the bone marrow of WT and KARAP^−/−^ mice after DSS exposure, and KEGG enrichment analysis of differential gene expression in neutrophils was established. (*B*) Representative ion chromatograms from targeted metabolomic analysis of tryptophan and its metabolites in bone marrow-derived neutrophils from WT and KARAP^−/−^ mice. (*C*) Orthogonal partial least squares-discriminant analysis (OPLS-DA) score plot demonstrating distinct metabolic profiles between WT and KARAP^−/−^ neutrophils. (*D*) Heatmap of differentially abundant metabolites in neutrophils. (*E*) Z-score plot of differential metabolites based on relative abundance. (*F*) Statistical comparison of Kyn levels between groups. (*G*) KEGG pathway analysis of the differential metabolites. (*H*) Enzyme-linked immunosorbent assay measurement of Kyn levels in neutrophils. (*I*) Coimmunoprecipitation (CoIP) and Western blot analysis of the interaction between KARAP and IDO1 in mouse neutrophils. (*J*) Immunofluorescence staining of AhR localization in neutrophils. Scale bar, 20 μm. ∗*P* < .05, ∗∗*P* < .01. DAPI, 4′,6-diamidino-2-phenylindole; FC, fold change.

### Killer Cell Activating Receptor–Associated Protein–Deficient Neutrophils Alleviate Mucosal Inflammation in Dextran Sulfate Sodium–Induced Colitis

To further investigate the immunomodulatory role of KARAP-mediated neutrophils in colitis, we established a DSS-induced colitis model in WT mice and then treated them intravenously with either WT or KARAP^−/−^ neutrophils. Mice receiving KARAP^−/−^ neutrophils exhibited significantly less body weight loss (Figure 10*A*). On the seventh day, all mice were sacrificed. We found that the colon length of mice treated with KARAP^−/−^ neutrophils was significantly longer than those of mice treated with WT neutrophils (Figure 10*B*). Pathologic analysis confirmed this protective effect, showing reduced inflammatory cell infiltration and lower pathologic scores in mice treated with KARAP^−/−^ neutrophils (Figure 10*C*). Consistently, we found that the number of neutrophils was significantly lower in the peripheral blood of mice treated with KARAP^−/−^ neutrophils than that in mice treated with WT neutrophils after DSS exposure (Figure 10*D*). At the molecular level, the expression of proinflammatory cytokines (eg, IL1β, IL6, and TNF-α) was significantly decreased in intestinal mucosa from mice treated with KARAP^−/−^ neutrophils compared with that from mice treated with WT neutrophils (Figure 10*E*). Furthermore, the levels of the tight junction proteins ZO-1, Occludin, and E-cadherin were significantly higher in intestinal mucosa from mice treated with KARAP^−/−^ neutrophils than in those of mice treated with WT neutrophils (Figure 10*F*). Therefore, these results demonstrate that KARAP-deficient neutrophils alleviate intestinal mucosal inflammation in DSS-induced colitis.

**Figure 10:**
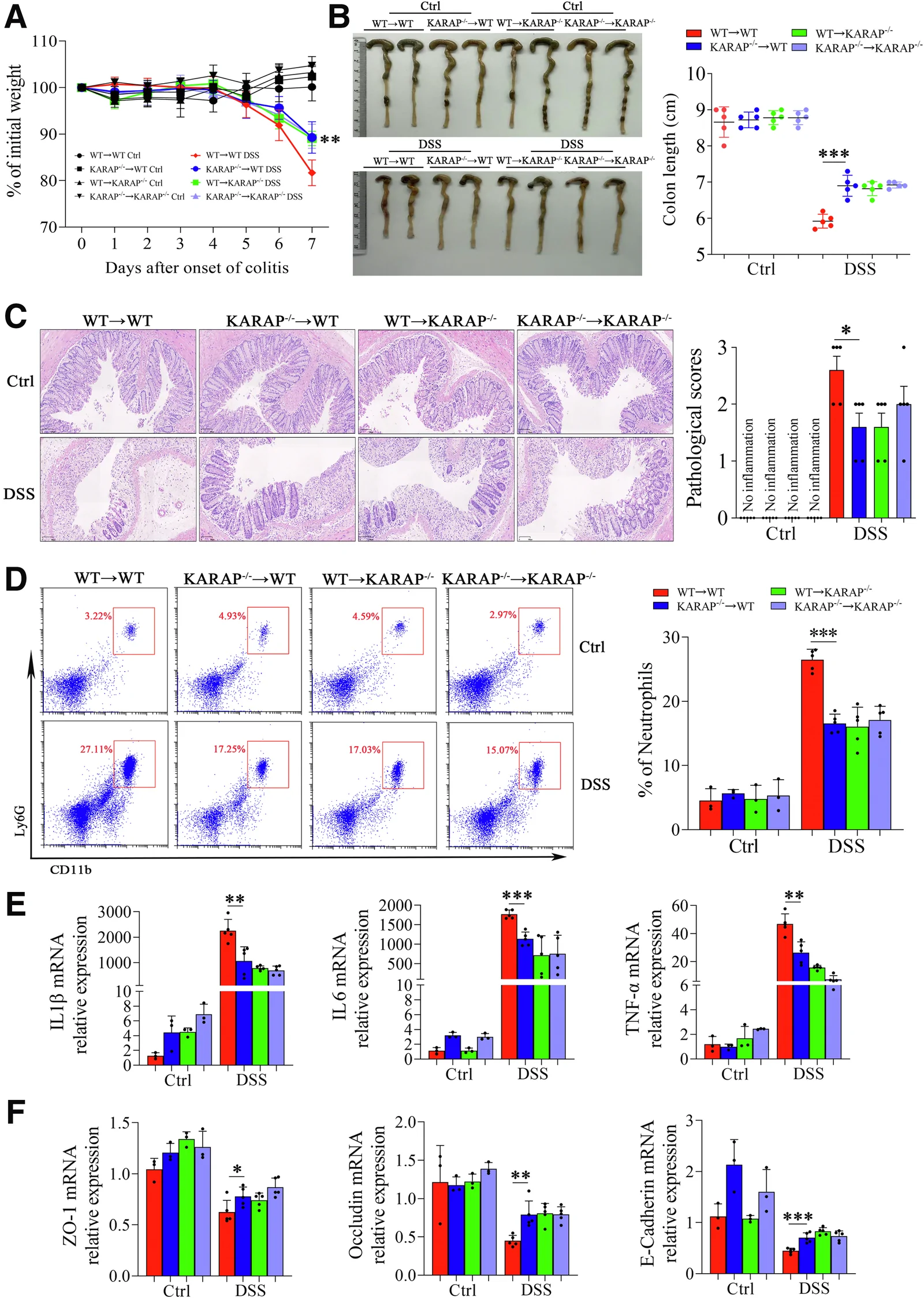
**KARAP-deficient neutrophils alleviate mucosal inflammation in DSS-induced colitis mice.** The colitis model was established in WT and KARAP^−/−^ mice by 2.5% DSS in drinking water, which were injected with WT or KARAP^−/−^ neutrophils (5 × 10^6^/mice) via the tail vein. (*A*) Body weight changes shown as percentage of initial weight in DSS-treated WT and KARAP^−/−^ mice that received tail vein injections of either WT or KARAP^−/−^ neutrophils. (*B*) The length of colons is shown. (*C*) Representative hematoxylin and eosin–stained colon sections and corresponding histopathologic scores. Scale bar, 50 μm. (*D*) Flow cytometric quantification of neutrophils in peripheral blood. (*E* and *F*) qRT-PCR analysis of (*E*) proinflammatory cytokines and (*F*) tight junction components in intestinal epithelial cells. ∗*P* < .05, ∗∗∗*P* < .001. mRNA, messenger RNA.

## Discussion

The recurrent and remitting characteristics of UC pose a significant challenge in clinical management. Neutrophils and related biomarkers of histopathologic activity offer valuable insights into diagnosing and predicting the clinical course of UC.^24^, ^25^, ^26^ Previous studies have proved that KARAP is widely expressed on the surface of neutrophils. In our study, we demonstrate that KARAP expression is elevated in the intestinal mucosa and neutrophils of patients with UC, which is positively correlated with disease activity. KARAP deficiency alleviates intestinal inflammation by promoting anti-inflammatory immune responses and promoting the intestinal mucosal barrier. Additionally, IL22 improves the intestinal mucosal barrier through a mechanism dependent on IDO1–Kyn, which promotes the nuclear translocation of AhR in neutrophils.

Previous studies have found that the expression of KARAP is significantly elevated in patients with rheumatoid arthritis and osteoarthritis and is closely related to disease activity.^27^ KARAP-deficient mice exhibit resistance to LPS-induced shock and bacterial septicemia, associated with attenuated systemic levels of TNF-α and IL6.^28^ Mice with KARAP gene expression deficiency are less likely to develop experimental autoimmune encephalomyelitis.^29^ KARAP can promote the activation and maturation of dendritic cells, enhance their migration ability, and induce upregulation of CD86 and CD40 molecules by activating ERK.^30^ KARAP can recruit downstream ZAP70 to form a complex, further activating the inflammatory signaling pathway, promoting the release of cytokines IL1β, IL6, TNF-α, and chemokines, ultimately promoting neutrophil degranulation and phagocytic activity and exerting an inflammatory amplification response.^31^ Despite these findings, the precise role of KARAP in UC remains unclear. In this study, KARAP expression was increased in the inflamed colonic mucosa of patients with UC and is highly expressed in neutrophils. Additionally, increased expression of KARAP in UC was positively correlated with disease activity. Moreover, KARAP deficiency alleviated mucosal inflammation in DSS-induced colitis in mice.

Neutrophils play a crucial role in maintaining intestinal epithelial homeostasis by releasing various cytokines in UC, among these cytokines, TNF-α, IL6, IL1β, and IL13 play important role in promoting the migration and activation of inflammatory cells, as well as contributing to damage to the intestinal barrier integrity.^8^ A correlation exists between elevated intestinal inflammation and the production of multiple cytokines. Moreover, neutrophils are also involved in eliminating invading pathogens and maintaining the integrity of the intestinal mucosal barrier. In our study, inhibition of KARAP in neutrophils markedly decreased the levels of TNF-α, IL1β, and IL6.^32^ KARAP-deficient mice displayed a marked decrease in intestinal mucosal inflammation caused by DSS assault. We previously observed a significant increase in reactive oxygen species, antimicrobial peptides, and NET from CD177^+^ neutrophils during intestinal mucosal inflammation in patients with inflammatory bowel disease.^11^ In the current study, KARAP suppression in neutrophils resulted in enhanced phagocytosis and bactericidal capacity, indicating its critical role in upregulating inflammatory immune responses. Therefore, the inhibition of KARAP expression during colitis may contribute to a reduction in inflammatory responses of neutrophils.

Damage or disruption of the intestinal mucosal barrier can result in inflammation and development of intestinal mucosal inflammation in UC.^33^ When the epithelial barrier is compromised, the gut microbiota infiltrates the lamina propria, triggering abnormal immune responses.^34^^,^^35^ Notably, recent studies have demonstrated that neutrophils can produce IL22, which subsequently enhances the intestinal mucosal barrier.^11^ In our research, KARAP^−/−^ neutrophils produced high levels of IL22, which further promotes tight junction formation in intestinal epithelial cells and fortifies the integrity of the intestinal epithelial barrier. Furthermore, elevated IL22 levels are primarily mediated through the AhR pathway in KARAP^−/−^ neutrophils, inhibition of AhR significantly reduces IL22 expression in KARAP knockout neutrophils, and suppression of AhR expression or neutralization of IL22 in KARAP^−/−^ mice exacerbated intestinal mucosal inflammation and destroyed intestinal mucosal barrier functions. Thus, KARAP deficiency improves intestinal mucosal barrier dependent on the AhR–IL22 pathway in neutrophils. However, the KARAP–AhR–IL22 pathway in vivo still requires further verification, and simultaneously, it cannot be ruled out that KARAP deficiency may lead to changes in AhR–IL22 levels in other cells, particularly in other myeloid cells, thereby affecting intestinal homeostasis. Further experimental investigation is still required.

KEGG enrichment analysis revealed that the differentially expressed genes in KARAP^−/−^ neutrophils were significantly associated with tryptophan metabolism. Tryptophan metabolism primarily involves 3 metabolic pathways: Kyn, 5-hydroxytryptamine, and indole. The predominant metabolic pathway for tryptophan is the Kyn pathway,^36^, ^37^, ^38^ through which over 95% of tryptophan is degraded into various bioactive compounds. Tryptophan 2,3-dioxygenase, IDO1, and IDO2 are critical rate-limiting enzymes in the Kyn pathway, which are reported to play a significant role in inflammation, immune responses, and excitatory neurotransmission.^39^^,^^40^ In our study, KARAP could interact with IDO1 directly and inhibit IDO1 expression and Kyn levels in neutrophils. Numerous tryptophan metabolites, including Kyn, facilitate the binding and activation of the AhR, which in turn induces the expression of downstream cytokines such as IL22 and IL17, thus regulating intestinal homeostasis.^41^, ^42^, ^43^ IDO1 serves as the metabolic enzyme responsible for the production of Kyn. In our study, the inhibition of IDO1 decreased the nuclear translocation of AhR in KARAP^−/−^ neutrophils. Consequently, the deficiency of KARAP in neutrophils led to increased levels of IDO1 and Kyn, which further enhanced the nuclear entry of AhR, thereby promoting IL22 production and contributing to the maintenance of the intestinal barrier.

In summary, our data demonstrate that KARAP inhibition is of great importance in protecting against intestinal mucosal inflammation, potentially through the IDO1–Kyn-mediated AhR–IL22 pathway in neutrophils. Therefore, our study underscores the critical role of KARAP in mucosal inflammation by regulating inflammatory response and integrity of the intestinal mucosal barrier in UC, positioning KARAP as a promising therapeutic target for UC.

## Materials and Methods

### Ethics Approval and Consent to Participate

All human studies were carried out with approval from institutional ethics committees for clinical research of Affiliated Hospital of Jining Medical University (Approval No. 2021C087). The animal ethics permit for this study was approved by the Affiliated Hospital of Jining Medical University (Approval No. 2022B101).

### Patients

Colon biopsy and EDTA-anticoagulated peripheral blood samples were collected from patients with UC and HCs at the Department of Gastroenterology, Affiliated Hospital of Jining Medical University between February 2021 and May 2025. All participants provided written informed consent prior to sample collection. UC diagnosis was confirmed based on standard clinical, radiologic, endoscopic, and histologic criteria.^12^ Disease severity was assessed using the Mayo index score, and endoscopic activity was graded with the Ulcerative Colitis Endoscopic Index of Severity.^44^ This study was approved by the Institutional Review Board for Clinical Research of the Affiliated Hospital of Jining Medical University.

### Bioinformatics Analysis

The dataset GSE109142 was downloaded from the Gene Expression Omnibus database, which includes RNA-seq transcripts per million counts from rectal biopsy tissues of patients with UC. Data were processed using R software (version 4.3.1). Based on the Mayo endoscopic score, disease severity was categorized as normal or mild (score < 6), moderate (score 6–10), and severe (score ≥ 11). Patients were stratified into low, intermediate, and high KARAP expression groups according to the lower and upper quartiles. A heatmap was generated using the ComplexHeatmap package in R. The neutrophil signature score was calculated as the mean of scaled log2-transformed expression values of constitutive neutrophil markers (NCF1, CSF3R, S100A8, S100A9, FCGR3B). Pearson’s correlation analysis was performed to assess the relationship between the neutrophil score and KARAP expression.

### Isolation of Neutrophils

Human peripheral blood neutrophils from patients with UC and HCs were isolated using Ficoll-Hypaque (GE Healthcare) density gradient centrifugation and EasySep Direct Human Neutrophil Isolation Kit (STEMCELL). Neutrophil purity was confirmed by flow cytometry, with >95% of cells being CD66b^+^. Mouse bone marrow neutrophils were harvested from femurs and tibias. After red blood cell lysis, neutrophils were isolated using the EasySep Mouse Neutrophil Enrichment Kit (STEMCELL). Purity was confirmed by flow cytometry, with >95% of cells being CD11b^+^Ly6G^+^.

### Isolation of Immune Cell Subsets

Peripheral blood mononuclear cells were isolated from EDTA-anticoagulated blood of HCs by density gradient centrifugation with Ficoll-Paque. CD4^+^ T cells were isolated from peripheral blood mononuclear cells by positive selection using antihuman CD4 particles and a cell separation magnet (BD). Cell purity was confirmed to be >90% by flow cytometry. Similarly, CD14^+^ monocytes, CD8^+^ T cells, and CD20^+^ B cells were isolated using magnetic bead-based separation kits.

### Single-Cell RNA Sequencing

Single-cell RNA-seq was performed by OEbiotech using the Chromium 10X Genomics platform and the Chromium Single Cell 3' Kit v3. Briefly, single-cell suspensions were loaded onto a Chromium Controller to generate gel beads in emulsion. Within the gel beads in emulsion, cells were barcoded and reverse-transcribed to generate complementary DNA, which was then amplified and used for library construction.

### RNA Sequencing Analysis

Neutrophils were isolated from the bone marrow of WT and KARAP^−/−^ mice. Total RNA was extracted, and its quality was assessed using a NanoDrop 2000 spectrophotometer (Thermo Fisher Scientific). Complementary DNA was synthesized using random N6 primers. RNA-seq was performed by the Beijing Genomics Institute on the BGISEQ-500 platform.

### Mice

KARAP^−/−^ mice were generated by GemPharmatech Co, Ltd. Specific pathogen-free WT and KARAP^−/−^ mice were housed under specific pathogen-free conditions with free access to autoclaved food and sterile water. All experimental mice were 8–10 weeks of age. The animal experiments were approved by the Institutional Animal Care and Use Committee of the Affiliated Hospital of Jining Medical University.

### Colitis Model in Mice

Colitis was induced by administering 2.5% DSS (molecular mass, 36,000–50,000, MP Biomedicals) in the drinking water for 7 consecutive days, followed by regular water. For each animal experiment, we utilized 5–8 mice per group. Mice were monitored daily for body weight, diarrhea, bloody stools, and survival. To investigate the role of AhR, DSS-treated WT and KARAP^−/−^ mice received intraperitoneal injections of either the AhR agonist FICZ (1 μg/mouse) or the AhR inhibitor CH223191 (10 mg/kg). In the neutrophil transfer experiments, WT mice were administered DSS starting on day 0. On day 1, neutrophils were isolated from both WT and KARAP^−/−^ mice and transferred into the model mice via tail vein injection, with a dosage of 5 × 10^6^ live cells per mouse. The mice continued to receive DSS treatment for a duration of 7 days, at which point their colonic conditions were analyzed.

### Coculture of Neutrophils With Intestinal Epithelial Cells

Mouse bone marrow–derived neutrophils were stimulated with LPS (200 ng/mL) for 3 hours. Caco-2 epithelial cells (ACC-169, Procellsystem) were cultured in RPMI 1640 medium supplemented with 10% fetal calf serum, 2 mM L-glutamine, and 100 U/mL penicillin and streptomycin. The cells were maintained in humidified incubators at 37 °C with an atmosphere of 5% carbon dioxide and 95% air. Caco-2 cells were cultured in the upper chamber of a Transwell system (0.4 μm pore size). After Caco-2 cells formed tight junctions, the stimulated neutrophils were added to the lower chamber and cocultured for 24 hours in the presence of IL22 (10 ng/mL), anti-IL22 antibody (1 μg/mL), FICZ (1 μM), or CH223191 (10 μM). The expression of tight junction proteins in Caco-2 cells was then assessed to evaluate epithelial barrier function.

### Targeted Detection of Tryptophan Metabolic Pathway

The tryptophan standard substance stock solution and the working standard solution were prepared accordingly. Metabolites were extracted from neutrophils isolated from WT and KARAP^−/−^ mouse bone marrow. Targeted analysis was performed using an ACQUITY UPLC HSS T3 column (2.1 × 150 mm, 1.8 μm; Waters) coupled with an AB Sciex ExionLC chromatograph and an AB Sciex 6500 Plus mass spectrometer.

### Phagocytosis Analysis

Neutrophils were isolated from the bone marrow of WT or KARAP^−/−^ mice and cocultured with FITC-labeled *E coli* under aerobic conditions at 37 °C for 6 hours. Phagocytosis was quantified by measuring FITC fluorescence intensity using flow cytometry.

### In Vivo Permeability Assay

Mice were fasted for 12 hours and subsequently gavaged with FITC-dextran (50 mg/kg). After 4 hours, the distribution of FITC-dextran was visualized using an IVIS Spectrum small animal imaging system (PerkinElmer). Blood was collected, and serum fluorescence intensity was measured with a fluorescence microplate reader (excitation: 480 nm, emission: 520 nm).

### Statistical Analysis

Data were analyzed using GraphPad Prism. Comparisons between 2 groups were performed with an unpaired Student *t* test. Multiple group comparisons were conducted using 1-way or 2-way analysis of variance followed by Tukey’s or Bonferroni’s post hoc test, as appropriate. Correlations were assessed using Spearman’s rank correlation coefficient. A *P* value of less than .05 was considered statistically significant.

## CRediT Authorship Contributions

Guiyuan Jin (Investigation: Supporting; Writing – original draft: Supporting)

Fengqin Zhu (Investigation: Equal; Writing – review & editing: Equal)

Fengxian Dai (Data curation: Equal; Methodology: Equal; Software: Equal)

Yan Wang (Data curation: Equal; Investigation: Equal; Methodology: Equal; Software: Equal)

Yun Chen (Data curation: Equal; Formal analysis: Equal; Software: Equal; Validation: Equal)

Yonghong Yang (Investigation: Equal; Methodology: Equal; Supervision: Lead; Writing – original draft: Equal; Writing – review & editing: Equal)

Zhanju Liu, MD, PhD (Supervision: Lead; Writing – original draft: Lead; Writing – review & editing: Lead)

Guangxi Zhou (Funding acquisition: Lead; Investigation: Lead; Writing – original draft: Lead; Writing – review & editing: Lead)

## Supplementary Material

Supplementary Video 2

Extended PDF
