# Supplementary material for: Killer Cell Activating Receptor–Associated Protein Exacerbates Intestinal Inflammation Through Promoting Kynurenine–Aryl Hydrocarbon Receptor–Dependent Neutrophil Activation in Ulcerative Colitis
Source: Cell Mol Gastroenterol Hepatol. 2026 Sep 24;21(1):101906. doi: 10.1016/j.jcmgh.2026.101906 (PMC13744314; doi:10.1016/j.jcmgh.2026.101906)

Supplementary Movies

AhR were observed in nucleus of WT and KARAP$^{-/-}$ neutrophils.

Neutrophils were treated with LPS (200 ng/mL) for 3h, then, All AhR (green) expression was detected by immunofluorescence, while the nuclear entry of AhR (red) was analyzed by removing the extranuclear AhR using Arivis software.

Full-length uncropped Western blots.

Figure 2E

KARAP H3cit β -actin

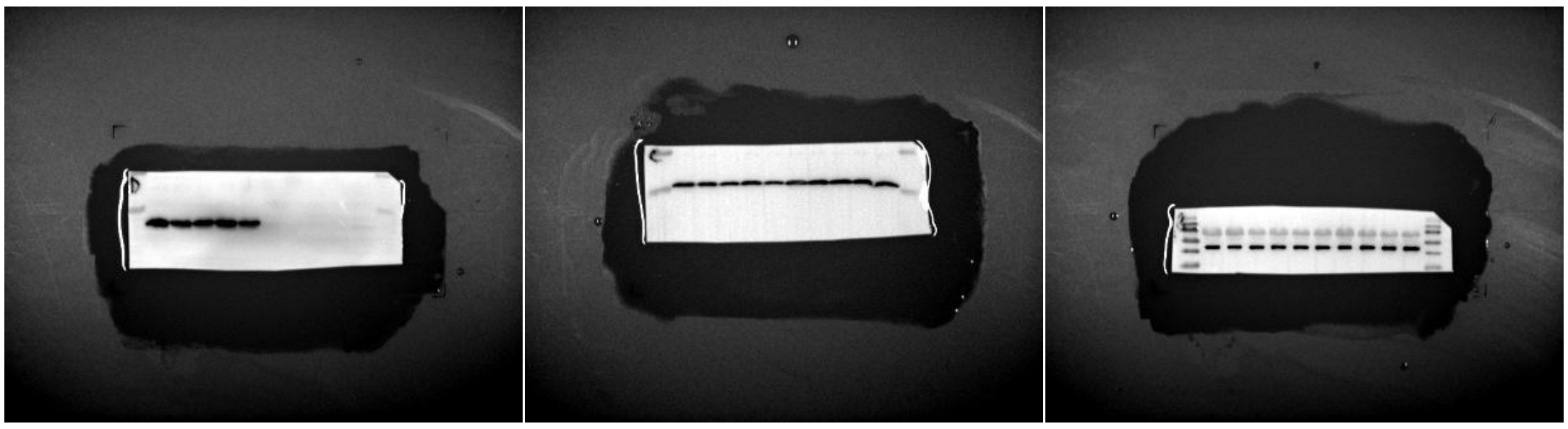

Figure 4D

ZO-1 Occludin β -actin

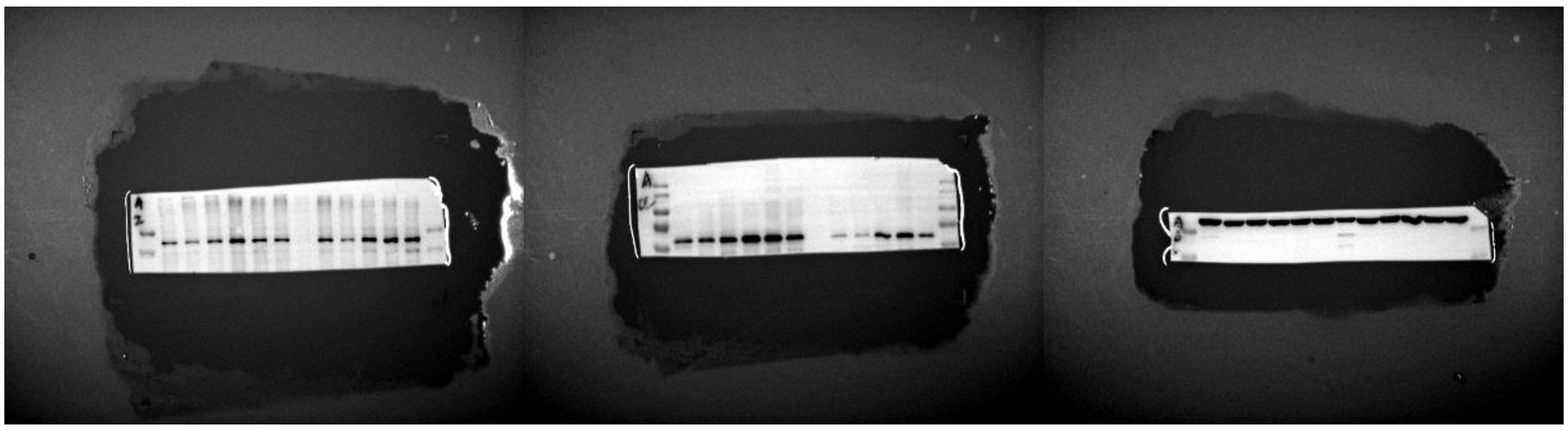

Figure 5D

ZO-1 β -actin

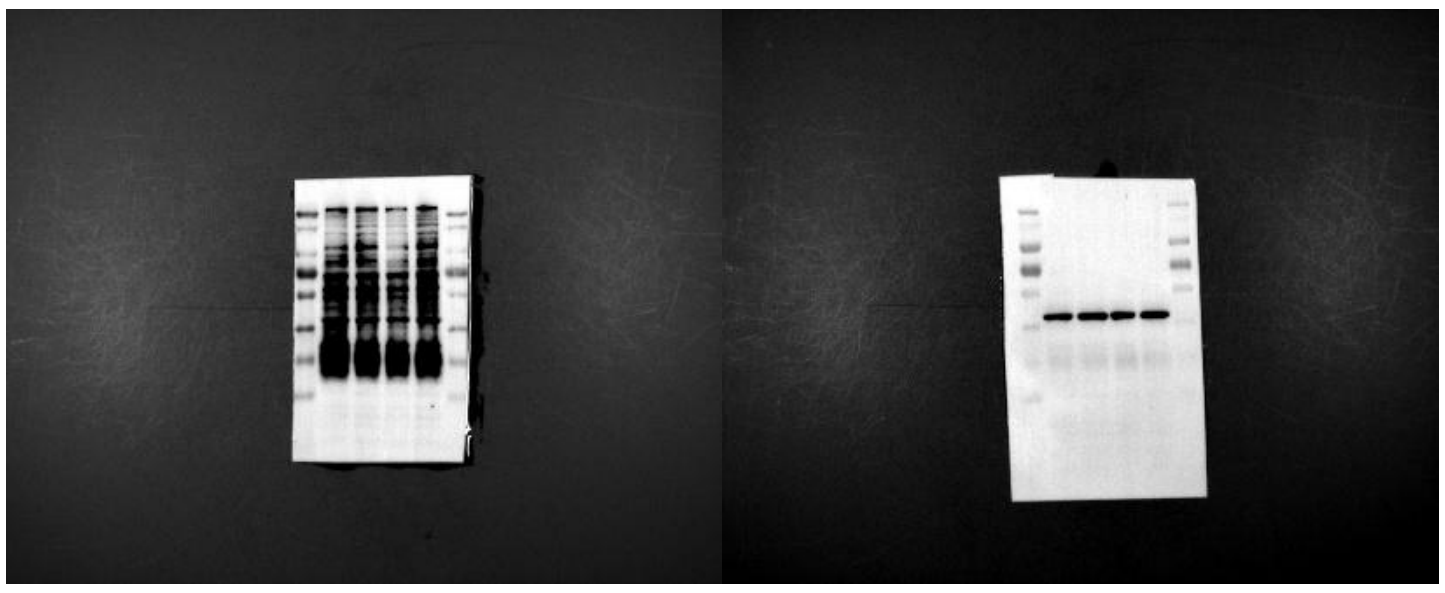

Figure 6B

AhR β -actin

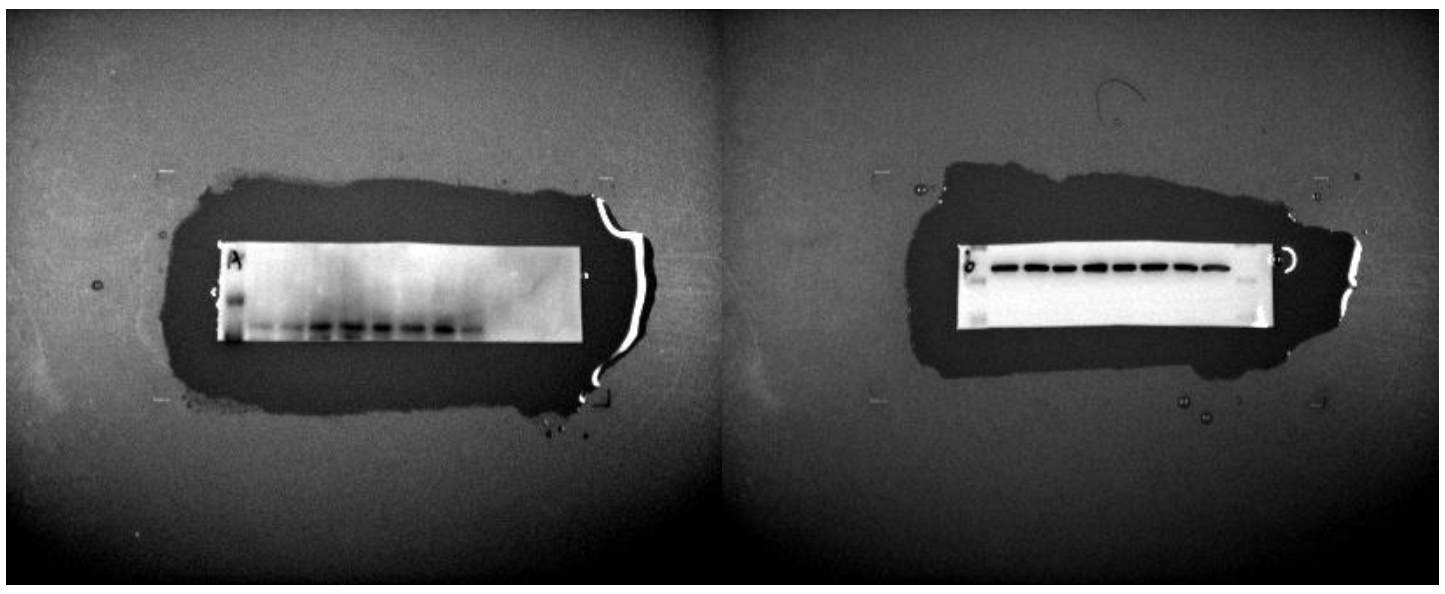

AhR(nuclear) Lamin B

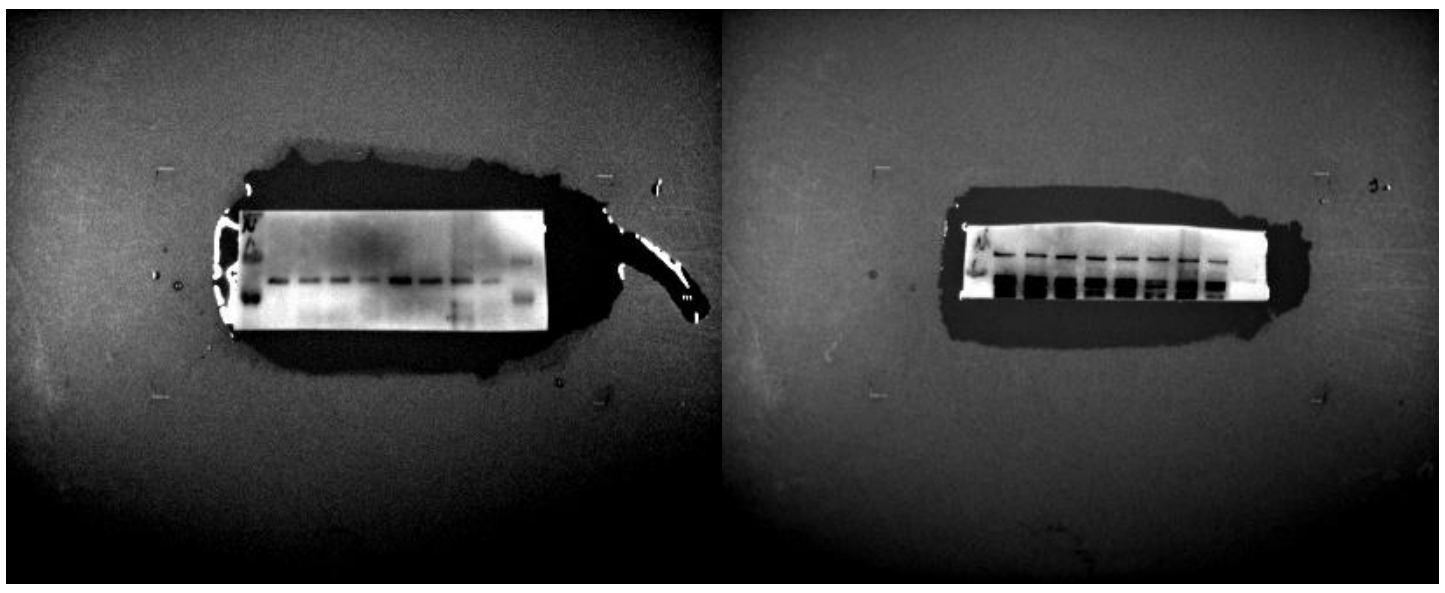

AhR(cytosol) β-actin

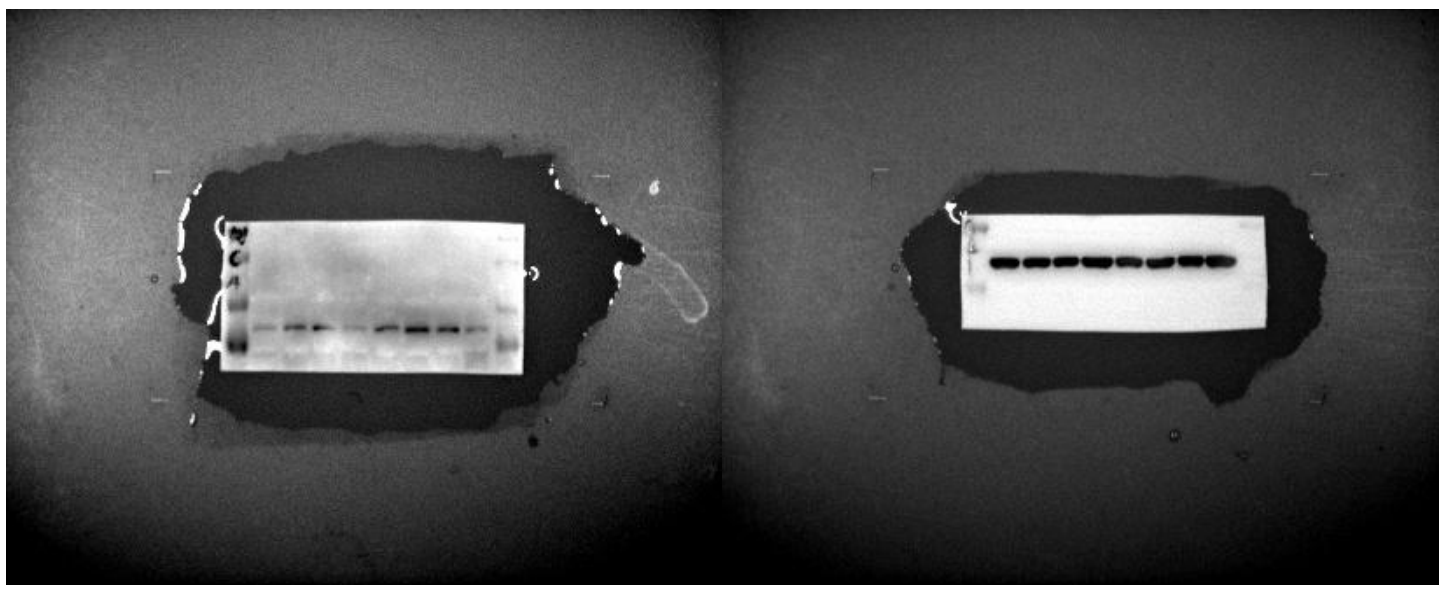

Figure 6G

ZO-1 Occludin β-actin

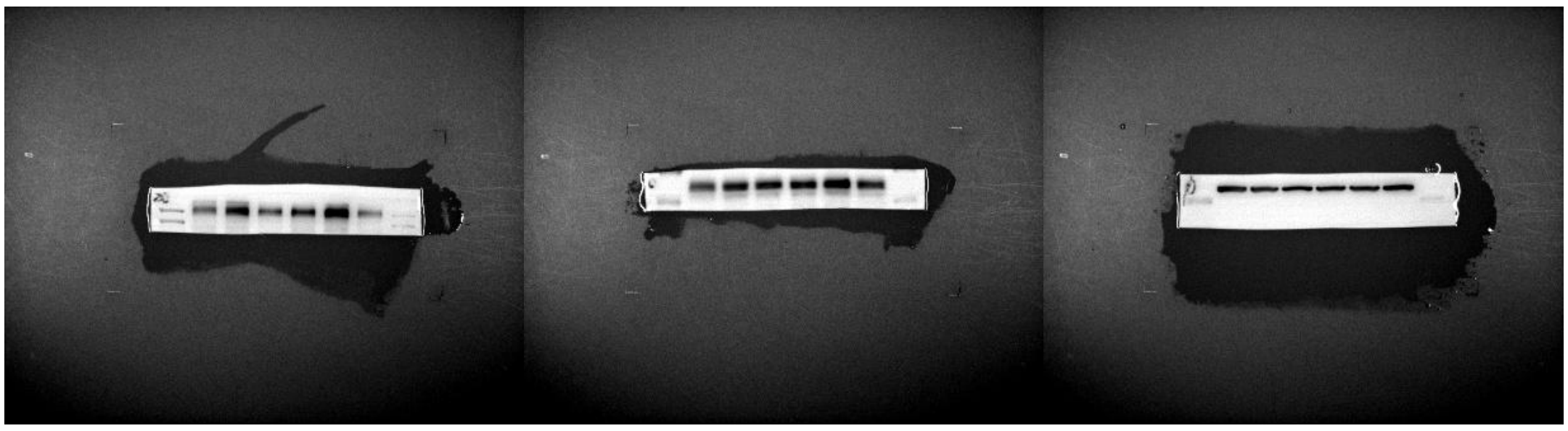

Figure 8B

IL-22 β-actin

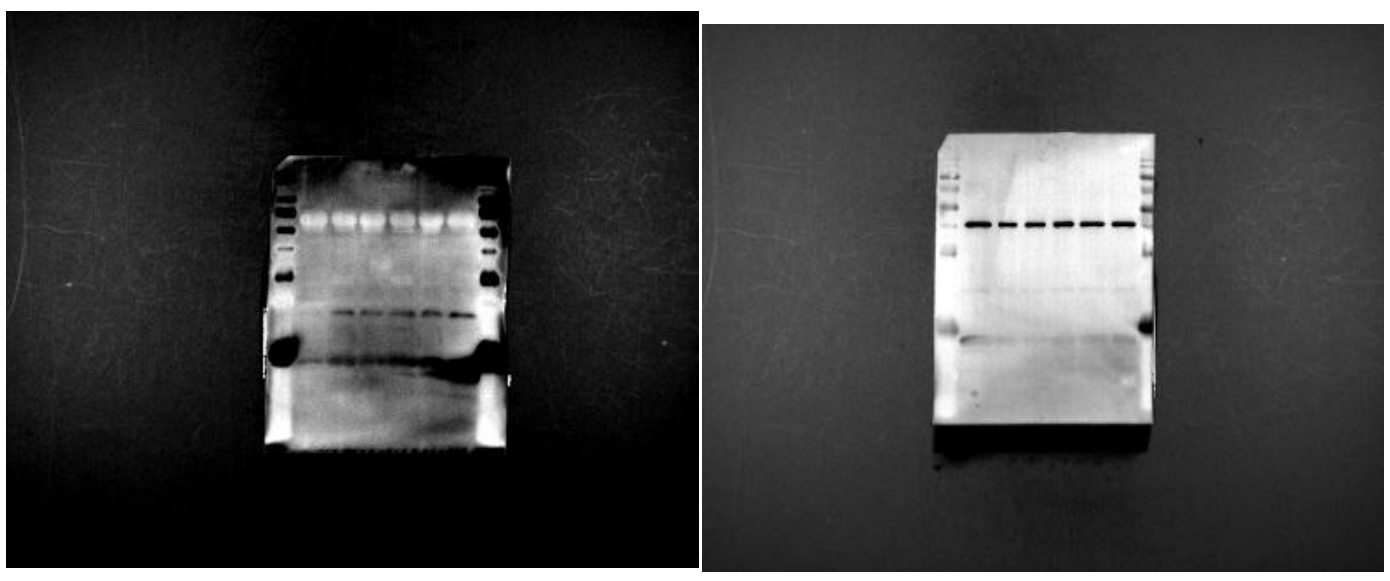

Figure 8H

ZO-1 Occludin β-actin

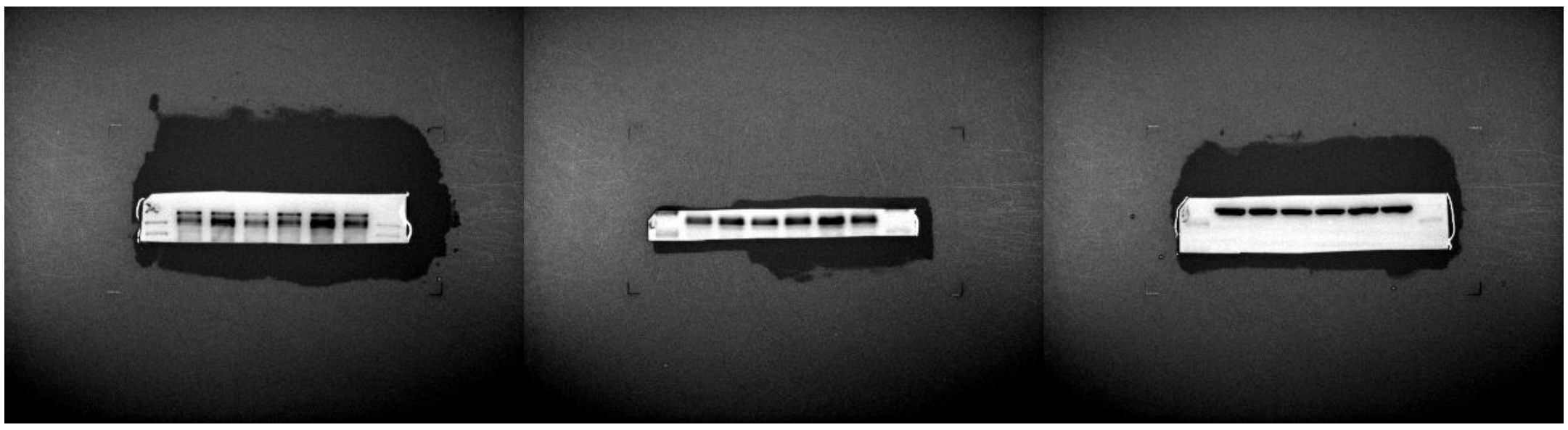

Figure 9I

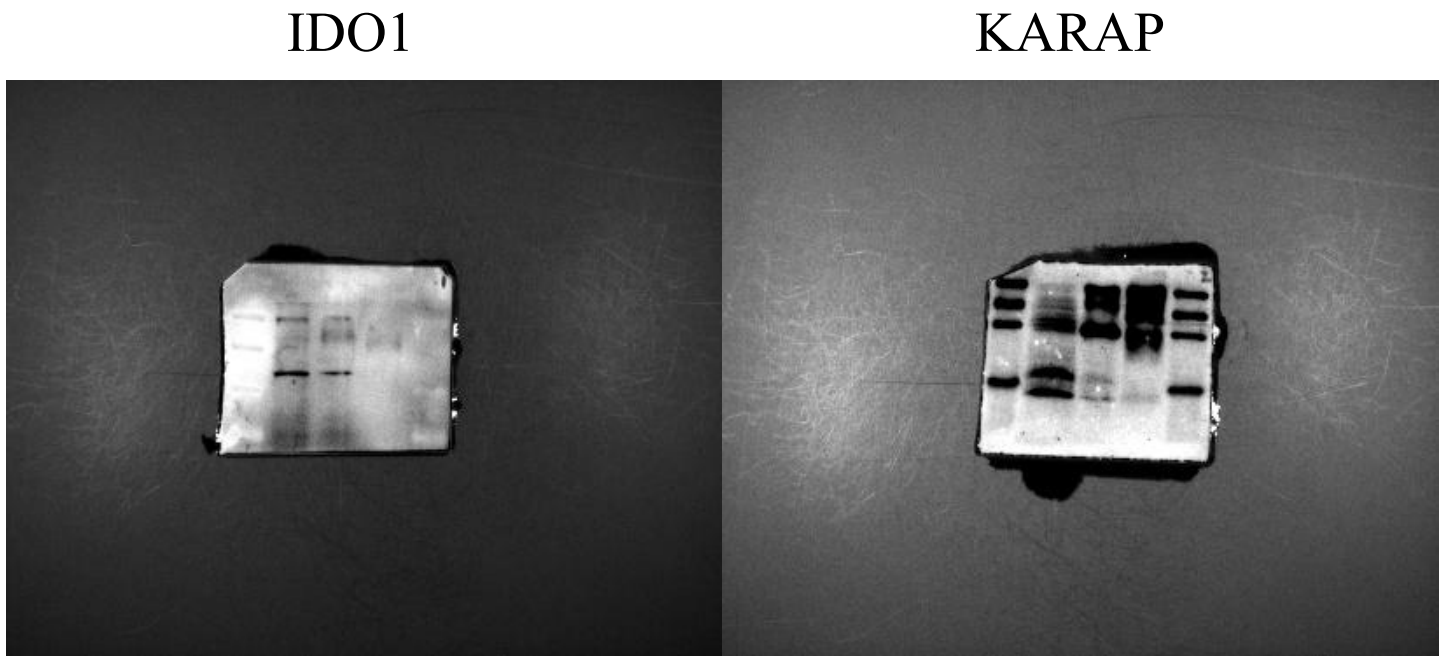

Supplement: Supplementary file 1 [file mmc1.pdf]
